# Differential gene expression and physiological changes during acute or persistent plant virus interactions may contribute to viral symptom differences

**DOI:** 10.1371/journal.pone.0216618

**Published:** 2019-05-03

**Authors:** Réka Pesti, Levente Kontra, Kenny Paul, Imre Vass, Tibor Csorba, Zoltán Havelda, Éva Várallyay

**Affiliations:** 1 Diagnostic Group, Department of Genomics, Agricultural Biotechnology Research Institute, National Agricultural Research and Innovation Centre, Gödöllő, Hungary; 2 Institute of Plant Biology, Biological Research Centre, Szeged, Hungary; 3 Virology Group, Department of Plant Biotechnology, Agricultural Biotechnology Research Institute, National Agricultural Research and Innovation Centre, Gödöllő, Hungary; 4 Plant Developmental Biology Group, Department of Plant Biotechnology, Agricultural Biotechnology Research Institute, National Agricultural Research and Innovation Centre, Gödöllő, Hungary; National University of Singapore, SINGAPORE

## Abstract

Viruses have different strategies for infecting their hosts. Fast and acute infections result in the development of severe symptoms and may cause the death of the plant. By contrast, in a persistent interaction, the virus can survive within its host for a long time, inducing only mild symptoms. In this study, we investigated the gene expression changes induced in CymRSV-, crTMV-, and TCV-infected *Nicotiana benthamiana* and in PVX- and TMV-U1-infected *Solanum lycopersicum* plants after the systemic spread of the virus by two different high-throughput methods: microarray hybridization or RNA sequencing. Using these techniques, we were able to clearly differentiate between acute and persistent infections. We validated the gene expression changes of selected genes by Northern blot hybridization or by qRT-PCR. We show that, in contrast to persistent infections, the drastic shut-off of housekeeping genes, downregulation of photosynthesis-related transcripts and induction of stress genes are specific outcomes with acute infections. We also show that these changes are not a consequence of host necrosis or the presence of a viral silencing suppressor. Thermal imaging data and chlorophyll fluorescence measurements correlated very well with the molecular changes. We believe that the molecular and physiological changes detected during acute infections mostly contribute to virus symptom development. The observed characteristic physiological changes associated with economically more dangerous acute infections could serve as a basis for the elaboration of remote monitoring systems suitable for detecting developing virus infections in crops. Moreover, as molecular and physiological changes are characteristics of different types of virus lifestyles, this knowledge can support risk assessments of recently described novel viruses.

## Introduction

High-throughput sequencing methods opened a new avenue for virus discovery [1]. However, more and more viruses are described each day, and assessing the importance and risk of these recently described viruses is very difficult. This basic question should be addressed in the future, not only for basic research but, more importantly, for decision makers who decide about quarantine regulations [2]. Detailed knowledge about different virus infections could help to identify key differences between acute and persistent virus infections [3].

Gene expression studies in virus-infected plants usually investigate events at early timepoints after the infection, i.e., just after the virus has entered the host cell. At this early timepoint, host antiviral responses are activated, and the fate of the infection is determined. If the plant is not a potential host, the virus cannot replicate. If the plant is a host of the virus, replication in the infected cell can happen, but thanks to the efficient resistance mechanisms of plants, most of the infections are confined to the infected cell, and the spread of the virus is blocked (reviewed recently in [4, 5]). In an interaction with a susceptible plant, the virus gets past this barrier and advances into the next stage of the infection, when it finally can move not only from cell to cell but also long distances, colonising the whole plant. During this process, other types of defence responses (such as antiviral RNA interference, RNAi) are activated [6]. Viruses can block RNAi mechanisms by expressing viral suppressor proteins to inhibit RNAi, and as a consequence, they can reach higher virus titres [7]. RNAi-linked molecular mechanisms and their potential role in disease induction are well described (reviewed in [8]); however, changes in the gene expression pattern of the host at this later step of infection still have not been fully characterized. With the availability of both host genomes and high-throughput methods such as microarray and RNA-sequencing (RNA-seq), an increasing number of studies investigate and characterize the molecular changes behind symptom development, usually in specific host-virus combinations [9–20].

During the virus infection, the virus alters the host metabolism to have its own genome replicated and to spread from cell to cell in the plant. A susceptible host can be sensitive or tolerant to a particular virus. In a sensitive host, the infection is usually acute: the virus accumulates to high levels and downregulates (shut-off) prominent housekeeping genes [21], resulting in severe disease symptoms that can even lead to the death of the plant within a short time. By contrast, in a tolerant host, the infection is persistent; the virus is also present in large concentrations but does not induce obvious symptoms, and therefore, the plant survives.

In our previous study, we observed that some plant-virus interactions are able to decrease expression of the important housekeeping genes, and this shut-off persists for several weeks [22]. By using *in vitro* run-on transcription experiments, we have also shown that shut-off manifests itself in the nucleus at the level of transcription [22]. We observed that the Cymbidium ringspot virus (CymRSV) and the crucifer-infecting tobacco mosaic virus (crTMV) showed very severe symptoms on *N*. *benthamiana*, and these infections finally culminated in the death of the plant. Potato virus X (PVX) showed intense chlorosis on *S*. *lycopersicum*, but necrosis never occurred, and the plants survived the infection. These infections were very fast, and intense downregulation of ribulose-1,5-bisphosphate carboxylase/oxygenase (Rubisco) and glyceraldehyde 3-phosphate dehydrogenase (GAPDH) could be detected as typical gene expression changes for acute infection. By contrast, turnip crinkle virus (TCV)-infected *N*. *benthamiana* and tobacco mosaic virus U1 (TMV-U1)-infected *S*. *lycopersicum* showed very mild symptoms of slight chlorosis. Neither the Rubisco nor the GAPDH levels were downregulated, which is typical for persistent infection [22].

During this study, our aim was to uncover common and specific trends of gene expression changes characteristic of acute or persistent viral infections. We used microarray, RNA-seq and Northern blot validation to follow the transcriptome changes. Employing diverse bioinformatics tools, we unravelled commonalities and specific features of acute and persistent infections. We have also shown that specific changes in physiological parameters are characteristic in acute infections. Based on these data, we propose that the molecular and physiological alterations may contribute to viral symptom development during acute infection.

## Materials and methods

### Plant materials and virus infection

Wild-type *Nicotiana benthamiana* [23] and *Solanum lycopersicum* (‘Kecskeméti jubileum’ cultivar of ZKI (www.zki.hu)) plants were used as systemic hosts. Plants were grown under a 16 h light/8 h dark regime at 21°C in soil. The *N*. *benthamiana* plants were infected with CymRSV, CymRSV19S (a p19 silencing suppressor mutant CymRSV), carnation Italian ringspot virus (CIRV), CIRV19S (a p19 silencing suppressor mutant CIRV), crTMV and TCV. The *S*. *lycopersicum* plants were infected with PVX and TMV-U1 strain. For infection, we used *in vitro* transcripts of the viruses (CIRV, CIRV19S, crTMV, PVX and TMV-U1) or purified virions (CymRSV, CymRSV19S, and TCV). The efficiency of the virus infection was tested either by EtBr gels or by Northern blot analysis of extracted RNA using radioactively labelled virus-specific probes. Cloned partial viral pieces were the templates for radioactive probe preparation. S19 Table contains a list of the primers used for cloning these viral sequences.

### Sample collection for high-throughput analysis

Infection experiments were performed three times, resulting in three biological replicates. In the case of *N*. *benthamiana*, we collected systemically infected leaves at 5 days post inoculation (dpi) (CymRSV-, crTMV- and mock-infected) and 11 dpi (TCV-infected) for microarray analysis and further validation. RNA was extracted from 10 plants/infection in three biological replicates (S1 Fig). In the case of *S*. *lycopersicum*, mock- and virus-infected ‘Kecskeméti jubileum’ variety at 14 dpi was used for RNA-seq and further validation (S2 Fig). Leaf samples were collected from the second leaf level from the bottom from four plants/infection in three biological replicates. RNA was immediately extracted from the samples, or the leaves were stored at -70°C.

### Total nucleic acid and RNA isolation

Total RNA was extracted [23] from the mock- or virus-infected (systemically infected) leaves. Briefly, frozen plant material was homogenized in an ice-cold mortar, suspended in 650 μl of extraction buffer (100 mM glycine, pH 9.0, 100 mM NaCl, 10 mM EDTA, 2% SDS and 1% sodium lauroylsarcosine) and mixed with an equal volume of phenol, centrifuged for 5 minutes. The aqueous phase was treated by equal volumes of phenol, chloroform, and isoamyl-alcohol (25:24:1), and after subsequent treatment with chloroform:isoamyl-alcohol (24:1), it was precipitated with ethanol and then resuspended in sterile water. Sample quality and viral presence were examined on a 1.2% agarose gel and by Northern blot analysis. This total nucleic acid was used for the Northern blot experiments. For microarray analysis and RNA-seq, the samples were further purified to eliminate DNA and improve the sample quality. Total nucleic acid extracts obtained were stored at −70°C until used.

### Microarray and bioinformatics analysis

For microarray analysis, total nucleic acid extracts of the pooled *N*. *benthamiana* samples (10 samples/infection in 3 biological replicates) were further purified by Tri Reagent (Sigma) and treated with DNase (Thermo Scientific). The concentration of the purified DNA-free RNAs was determined by NanoDrop, and their quality was further checked by Agilent scanner, FE SW 9.5. Samples with adequate quality were used for microarray hybridization using the Agilent Tobacco Gene Expression Microarray 4x44K array. Expression profiles of the virus-infected samples were compared to those of the control plants. Raw data were deposited into NCBI-GEO: GSE113774/ GPL21056. Mean normalized expression values for each probe were calculated (3 biological replicates for 3 virus and mock infections), and these normalized results for all samples were also deposited into NCBI-GEO. Genes showing statistically significant changes in response to infection by crTMV, CymRSV and TCV were identified using corrected *p* value assignments <0.05 that were derived from unpaired T-test variance analysis and followed by multiple testing correction by the Benjamini–Hochberg procedure. Changes in gene expression were calculated as normalized fold changes, and changes greater than twofold were analysed (S1 Table).

### RNA-sequencing and bioinformatics analysis

For RNA-seq, total nucleic acid extracts of pools derived from *S*. *lycopersicum* plants (4 samples/infection in 3 biological replicates) were purified with RNAzol. Their quality was checked on a Bioanalyser. Libraries were prepared with Illumina TruSeq Stranded mRNA Library prep kit, and 50 base-pair single-end reads were sequenced on an Illumina HiScanSQ by the UD-GenoMed company. The third biological replicates were sequenced twice as a technical replicate; therefore, four fastq files containing results of the sequencing were deposited into NCBI-GEO: GSE113774/GPL24943. For quality control, the reads were checked with fastqc, ribosomal contaminations were removed with SortMeRNA [24], and adapter trimming and quality filtering were performed by Trimmomatic [25]. After quality control, the reads were aligned to the genome (Sly2.5 with ITAG2.4 annotation) and quantified, and a significant expressional difference was calculated using the Tuxedo protocol [26]. Only genes showing significantly different expression (FDR-adjusted *p* value less than 0.05) and twofold changes were considered (S2 Table). Heat maps were created to show the gene expression of several probes or genes using red colour for up- and green for downregulated genes. A detailed version of each heat map is provided as a supplementary Excel table containing log2-fold change values with their corresponding *p* values.

### Northern blot analysis of host endogenous genes

*N*. *benthamiana* and *S*. *lycopersicum* total RNA extracts were used for first-strand cDNA synthesis (RevertAid First Strand cDNA Synthesis Kit, Thermo Scientific) according to the manufacturer’s recommendation and using oligo dT. Target gene fragments were amplified from the produced cDNA by PCR using Taq polymerase (Thermo Scientific). The sequences of the oligonucleotides used as primers are listed in S11 and S12 Tables. The products of the reverse transcription PCR (RT-PCR) were purified by GenJet (Thermo Scientific). The gel-purified PCR products were ligated into pBlueScript plasmid (Promega). The sequences of the clones were confirmed by Sanger sequencing. For Northern blot analysis, 1–5 μg of total RNA were separated on formaldehyde-containing 1.2% agarose gels and blotted to Hybond-N membranes, as described previously [27]. Cloned fragments of the viruses and endogenous genes being studied were used as templates for probe preparation. Before probe preparation, the dNTPs were eliminated from the PCR product by gel filtration of the product on homemade G-50 Sephadex micro columns. Radioactively labelled random DNA probes were generated from these purified PCR products with a DecaLabel DNA Labeling Kit (Thermo Scientific). Northern blots were hybridized with these probes either in Church buffer (1% BSA, 1 mM EDTA, 0.25 M Na2HPO4, 7% SDS, pH 7.2), DIG Easy Hyb(Roche) or PerfectHyb Plus Hybridization buffer (Sigma-Aldrich), at 65°C, washed according to the manufacturer’s instructions and exposed to X-ray films. Signal intensity was calculated using the rRNA band of the EtBr-stained gel before blotting as a loading control.

### Quantitative RT-PCR analysis

When Northern blot analysis was not successful in detecting endogenous target transcripts, we used quantitative RT-PCR for validation of gene expression changes. For qRT-PCR, the total RNA isolated by the phenol-chloroform method was treated with DNase (Thermo Scientific), followed by purification using RNAzole (Sigma). Then, 3 μg RNA was used to prepare cDNA using RevertAid Reverse Transcriptase and a random oligo primer (Thermo Scientific). cDNA was used as a template for PCR, which used gene-specific primers (S11 and S12 Tables) and Power SYBR Green PCR Master Mix (Applied Biosystems) and was performed in a Rotor Gene 3000 real-time DNA detection system (Qiagen). Three biological replicates and three technical replicates were used per sample. For relative gene expression results, the delta-delta Ct was calculated. Ubiquitin showing stable expression changes in every infection in both hosts was used as an internal control.

### IR thermography

For thermography, we selected the third, fully developed young leaves from the apical shoots of *Nicotiana benthamiana* (CymRSV and crTMV on 4 dpi; TCV on 8 dpi) or *Solanum lycopersicum* (13 dpi for both viruses) plants. Thermographic images were taken using a Stirling-cooled infrared scanning camera (varioSCAN 3021 ST, Jenoptik, Jena, Germany). All plants under treatment were assessed by thermography, with 3 plant replicates per treatment. Leaf temperatures were analysed independently from each other [28, 29]. The mean leaf temperature per plant was used for statistical analysis (n = 3 to 5).

### Chlorophyll a fluorescence and Chl fluorescence imaging

Leaves of the third fully developed branch from the apical shoot were used for the study in both hosts. Plants were analysed at 7 dpi (in the case of CymRSV- and crTMV-infected *N*. *benthamiana*), at 11 dpi (in the case of TCV-infected *N*. *benthamiana*) and at 8 dpi (in the case of TMV- and PVX-infected *S*. *lycopersicum*) together with their appropriate controls. OJIP chlorophyll a fluorescence transients were measured by a Plant Efficiency Analyzer (Pocket Pea, Hansatech, UK). Prior to the OJIP measurements, plants were dark-adapted for 20 min under greenhouse conditions. Nine leaves from 3 independent plants/treatment were analysed to obtain data. Quenching analysis of Chl fluorescence was detected by the maxi-head of a PAM Chl fluorescence imaging system (Walz Effeltrich, Germany).

The chlorophyll fluorescence parameters (that determine photosynthetic performance) were measured or calculated as follows: the initial fluorescence yield was obtained in a dark-adapted state, when the reaction centres are open (Fo); maximal fluorescence yield, in a dark-adapted state, when the reaction centres are closed (Fm); maximal quantum yield of photosystem II photochemistry (Fv/Fm); electron transport rate at donor side of PSII (Area) reflecting the size of the plastoquinone pool; the amount of active reaction centres per absorption (RC/ABS); efficiency of water splitting complex on the donor side of PSII (Fv/Fo); probability of electron transport out of QA ((1-Vj)/ Vj, where Vj = (F2ms–Fo)/Fv); and performance index (potential) for energy conservation from photons absorbed by PSII to reduce the intersystem electron acceptors (PI) [30].

## Results and discussion

Based on viral symptoms and infection lifestyles, we employed a range of single-stranded RNA viruses to study whole transcriptome changes induced during acute or persistent infections. *N*. *benthamiana* plants were infected with CymRSV (Tombusviruses, acute), crTMV (Tobamoviruses, acute) and TCV (Tombusviruses, persistent), whereas *S*. *lycopersicum* plants were infected with PVX (Potexviruses, acute) and TMV-U1 (Tobamoviruses, persistent). CymRSV and crTMV infections on *N*. *benthamiana* plants resulted in a high level of virus accumulation in the systemic leaves at 5 dpi, which is associated with drastic downregulation of housekeeping gene mRNAs, as demonstrated by monitoring virus and GAPDH mRNA levels [22]. This acute phase of infection turns rapidly into necrosis and subsequent death of plants at 7–8 dpi. The acute PVX infection on *S*. *lycopersicum* is slower, with the virus reaching the systemic leaves at approximately 14 dpi, coinciding with the downregulation of housekeeping genes. Although severe disease symptoms appear, the plant can survive while exhibiting prolonged downregulation of housekeeping genes. TCV infection on *N*. *benthamiana* is slower than CymRSV and crTMV infections, with the virus reaching the systemic leaves at 11 dpi. In this case, the downregulation of housekeeping genes never occurs, and the infection never turns into the acute phase. TMV infection on *S*. *lycopersicum* shows the same characteristic pattern, with the virus reaching the systemic leaves at 14 dpi but never inducing downregulation of the housekeeping genes [22]. According to these results, we identified sampling timepoints in this current work to represent the earliest timepoints when viruses systemically infect their host plants and induce symptoms such as stunting and chlorosis, but only when necrotic reactions are not yet initiated (S1 and S2 Figs).

### Host transcriptomes are strongly affected during acute virus infections

**Fig 1:**
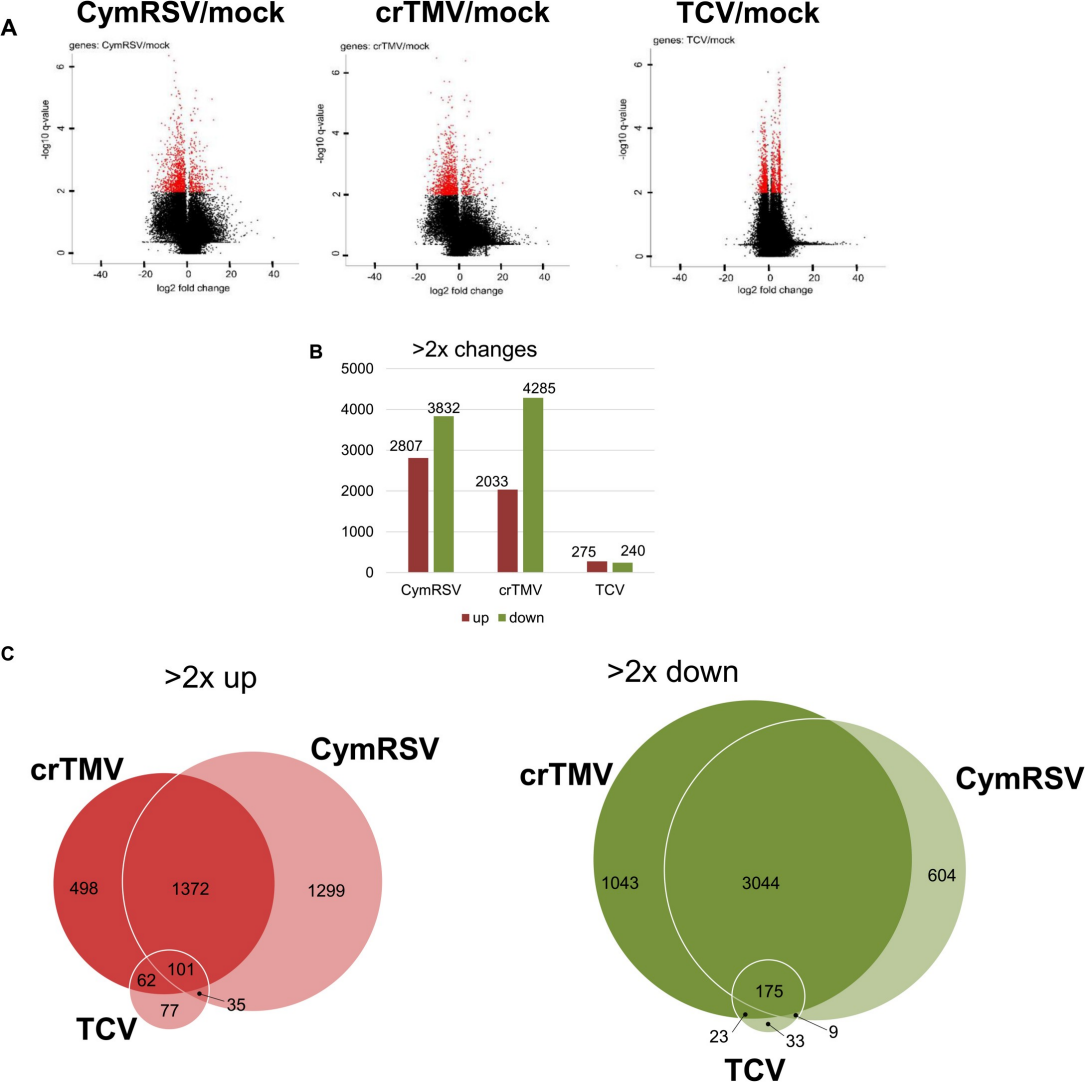
Gene expression changes in virus-infected *N*. *benthamiana* plants. Differentially expressed probes were obtained by comparing the gene expression of virus-infected and mock-treated samples. Fold changes in log2 were used to generate volcano plots. More than 2-fold changes and *p* values less than 0.05 were applied to identify the DEPs. (A) Volcano plots display log2-fold changes and *p* values. (B) Column diagram of the number of DEPs showing at least a 2-fold change. (C) Venn diagrams of DEPs identified in the virus-infected samples. Circle areas reflect the number of DEPs. All DEPs are listed with their characteristic parameters in S1 Table.

To unravel changes caused by CymRSV-, crTMV-, TCV (and mock) infection in *N*. *benthamiana* plants a microarray analysis was carried out (see Materials and Methods). To find differentially expressed genes, we collected all probes showing 2-fold changes with *p* values smaller than 0.05 in any of the virus-infected plants (listed in S1 Table). In CymRSV- and crTMV-infected plants, the number of differentially expressed probes (DEPs) was very high (6639 and 6318), while in TCV-infected samples, the number of DEPs was lower by approximately an order of magnitude (515 DEPs) (Fig 1A and 1B). Among these DEPs, 2807, 2033 and 275 probes were upregulated, whereas 3832, 4285 and 240 were downregulated (Fig 1B). Importantly, most of up- (1473) and downregulated (3219) probes were similar in the CymRSV- and crTMV-infected plants, suggesting common regulation (Fig 1C). These results show that acute infections (CymRSV and crTMV) severely alter the transcriptome of the host, whereas the persistent infection (TCV) has a limited impact.

**Fig 2:**
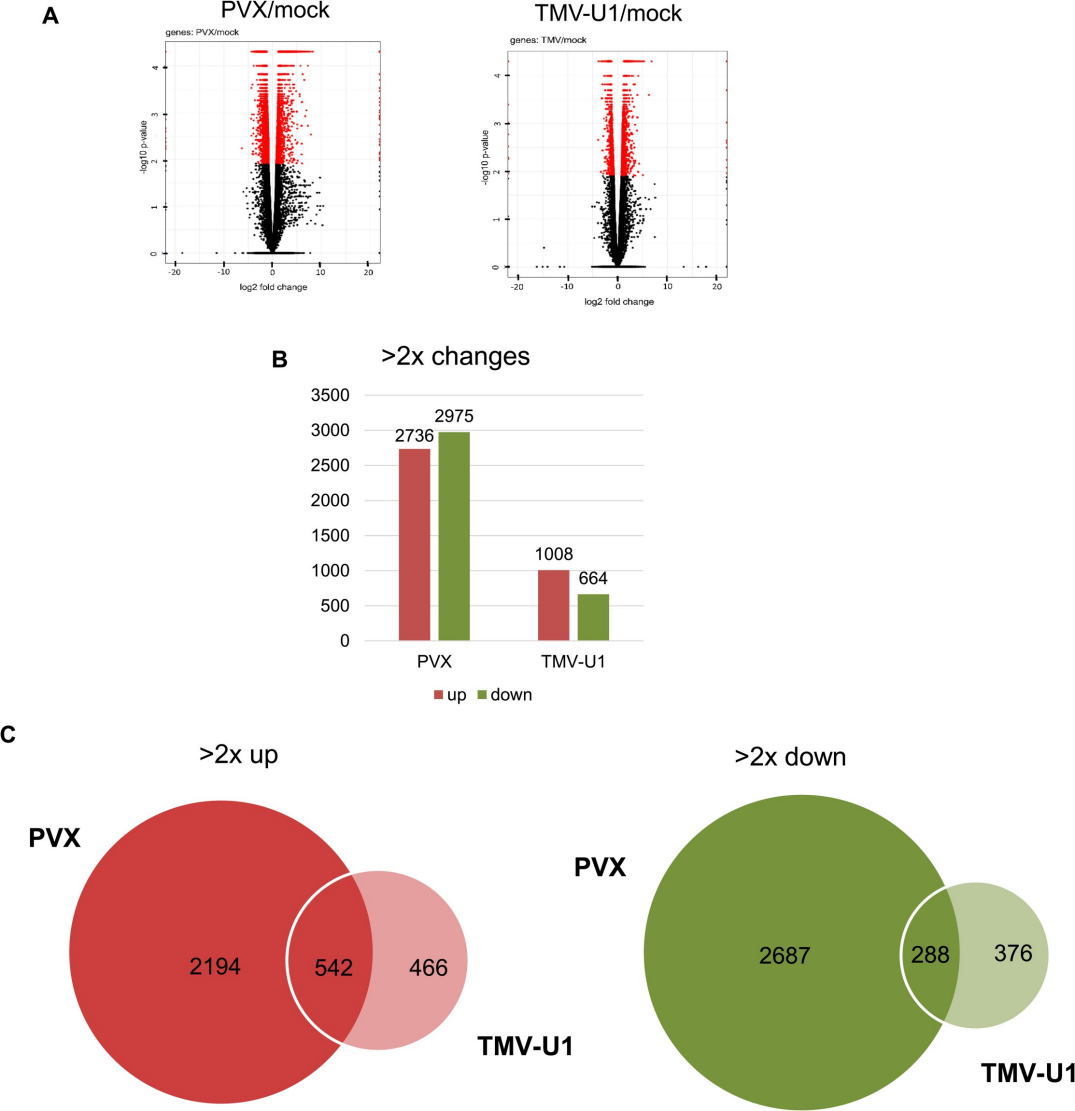
Gene expression changes in virus-infected *S*. *lycopersicum* plants. Differentially expressed probes were obtained by comparing the gene expression of virus-infected and mock-treated samples. Fold changes in log2 were used to generate volcano plots. Changes of more than 2-fold and *p* values less than 0.05 were applied to identify the DEGs. (A) Volcano plots display log2-fold changes and *p* values. (B) Column diagram of the number of DEGs showing at least a 2-fold change. (C) Venn diagram of DEGs identified in the virus-infected samples. Circle areas reflect the number of DEGs. All DEGs are listed with their characteristic parameters in S2 Table.

An RNA-seq analysis was carried out to study transcriptome changes during PVX and TMV-U1 infection in *S*. *lycopersicum* plants (see Materials and Methods). The differentially expressed genes (DEGs, 2-fold changes, p <0.05) observed during virus infection are listed in S2 Table. A total of 5711 DEGs were identified in the PVX-infected samples (vs mock), whereas in the TMV-infected samples, only 1672 DEGs were found. Among these, 2736 and 1008 DEGs were upregulated and 2975 and 664 DEGs were downregulated in PVX- and TMV-infected plants, respectively (Fig 2A and 2B). Only a small fraction of the DEGs (542 and 288) overlapped between the two infections (the majority of DEGs were present only in the PVX-infected plants) (Fig 2C).

Comparison of Volcano plots shows that the number of downregulated genes was much higher in *N*. *benthamiana* than that of upregulated genes, whereas in *S*. *lycopersicum*, this ratio was approximately 1:1 (Figs 1A and 2A). However, probes on the *N*. *benthamiana* chip could not be directly correlated to changes in the whole genome because some transcripts could be overrepresented, whereas probes for others could be absent. An imbalance of the genes represented on the chip could lead to an imbalance in the identified DEGs and not actually show their real ratio.

Therefore, in summary, acute infections cause severe transcriptome changes, whereas persistent infections have a moderate impact. The changes caused by acute and persistent infections in *N*. *benthamiana* and *S*. *lycopersicum* plants are consistent with each other.

### Photosynthesis-related transcript downregulation and stress-related transcript up-regulation occur specifically during acute infection

To reveal the molecular basis of acute and/or persistent infections, we analysed the functional distribution of the DEGs (annotated according to their Bin codes). The most differentially regulated genes common for acute and persistent infections (for all viruses tested) were the ones that participate in protein metabolism, followed by the DEGs of RNA regulation, signal processes and transport (S3 Fig). These changes were observed in both hosts. When we searched for DEGs, we found that transcripts that play role in photosynthesis were downregulated, whereas stress response DEGs were upregulated almost exclusively during acute (CymRSV, crTMV and PVX) infections and were typically absent in persistent (TCV and TMV-U1) infections (S4 Fig and S3–S6 Tables). Investigating the top 20 down- and upregulated genes in both hosts, we found the same situation: changes were more severe during acute infections (S7–S10 Tables). The most downregulated genes in *N*. *benthamiana* plants were almost the same during acute infections (CymRSV and crTMV), and the expression of these probes was unaltered during persistent (TCV) infection. The most upregulated genes in virus-infected *N*. *benthamiana* plants were stress-related genes and were very similar during CymRSV- and crTMV-infected tobacco but were unchanged in the TCV-infected tobacco (S8 Table).

The most downregulated DEGs in *S*. *lycopersicum* have diverse functions, except the cytochrome P450s, whose levels decreased in both PVX and TMV infections. Although some of the top 20 downregulated genes were similar, the extent of their downregulation was more severe during acute infection (PVX-infected tomato) (S9 Table). Pathogenesis-related proteins (PRs) and various transcription factors that play a role in hormone metabolism were among the most upregulated genes in the virus-infected tomato. In addition, in this case, the PVX-induced changes were even much higher compared to TMV-U1 (S10 Table). In summary, photosynthesis-related and stress-related transcripts are specifically affected during acute infection.

### Housekeeping-, stress- and some metabolism-related transcripts are differentially expressed during acute but not persistent infection

**Fig 3:**
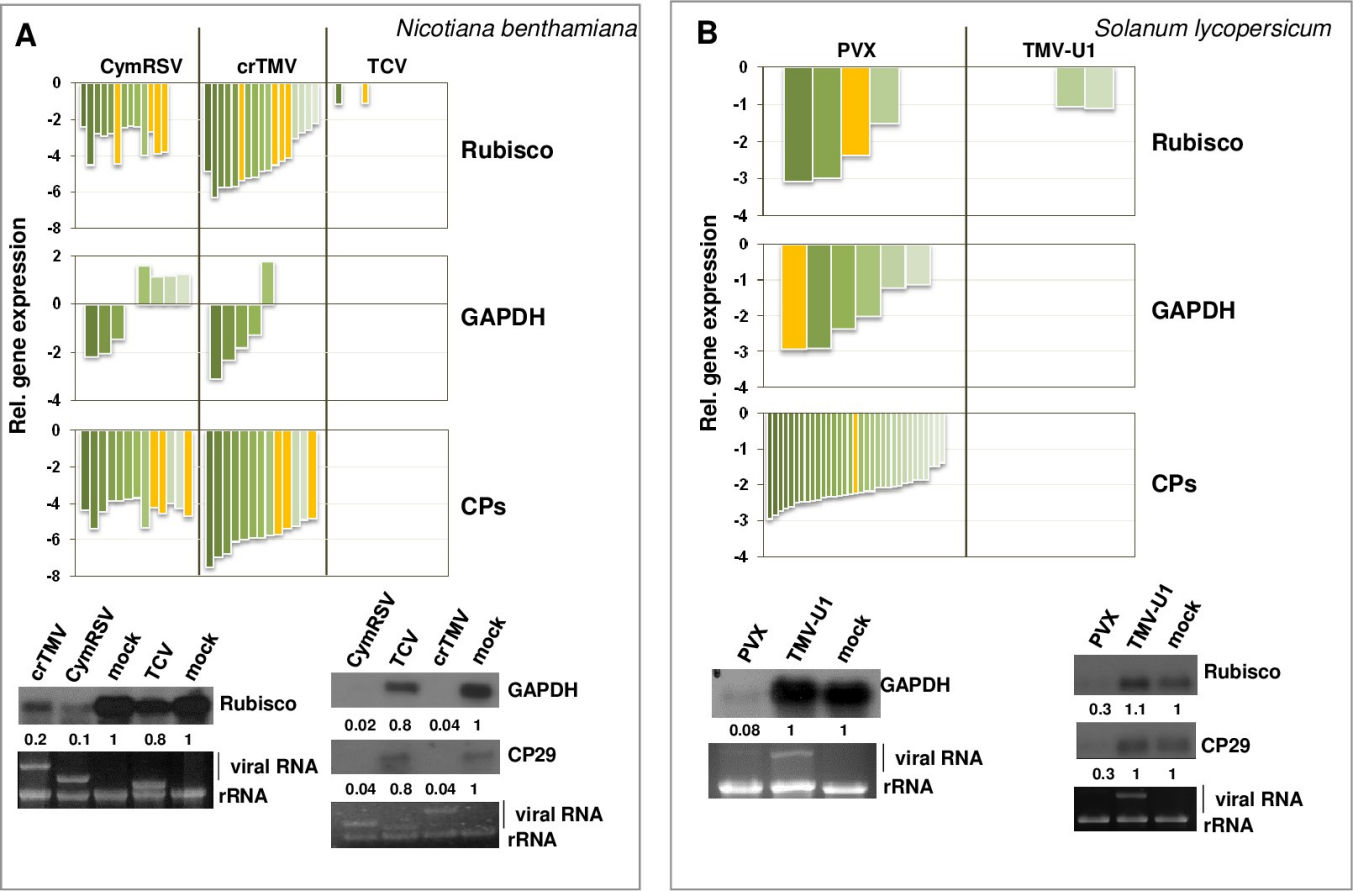
**Investigation of the gene expression changes of Rubisco, GAPDH and different chlorophyll binding proteins (CPs) in virus-infected *N*. *benthamiana* (A) and *S*. *lycopersicum* (B) plants.** The column diagram in the upper panel shows log2-fold changes of probes or genes specific for the investigated gene obtained by microarray analysis (*N*. *benthamiana*) or by RNA-seq (*S*. *lycopersicum*). The lower panel shows Northern blot hybridization using radioactively labelled probe specific for endogenous genes. Fold-change values are shown below each band (quantifications were normalized to mock controls). EtBr-stained gel served as a loading control. Yellow columns depict genes whose levels were validated by Northern blot hybridization. (The investigated *N*. *benthamiana* GAPDH probe was absent on the microarray chip).

In our previous work, we showed that, in systemic leaves of CymRSV-infected *N*. *benthamiana*, the level of GAPDH, tubulin, Rubisco, chlorophyll a/b binding protein (CP) 29 (CP29) and histone (H1E) transcripts were drastically downregulated, and the elongation factor (EF) 2 transcript was slightly decreased, whereas the glutathione S-transferase gene (GST) and the heat shock protein (HSP) 90 gene (HSP90) transcripts were increased [22]. We checked the expression levels of these genes in our microarray and RNA-seq data. Several Rubisco-, GAPDH- and CP-specific probes were present on the microarray chip. Their expression showed severe changes only during acute (CymRSV and crTMV) infections, and their expression was not altered during persistent (TCV) infection (Fig 3A upper panel). We validated the changes of the Rubisco, GAPDH and CP29 transcripts by Northern blot analysis (Fig 3A bottom panel). The RNA-seq results from *S*. *lycopersicum* plants showed that Rubisco, GAPDH and CP RNA levels decreased during the PVX infection but were not altered or only slightly so during TMV-U1 infection (Fig 3B upper panel). Validation of their levels by Northern blot analysis showed the same trend (Fig 3B bottom panel).

**Fig 4:**
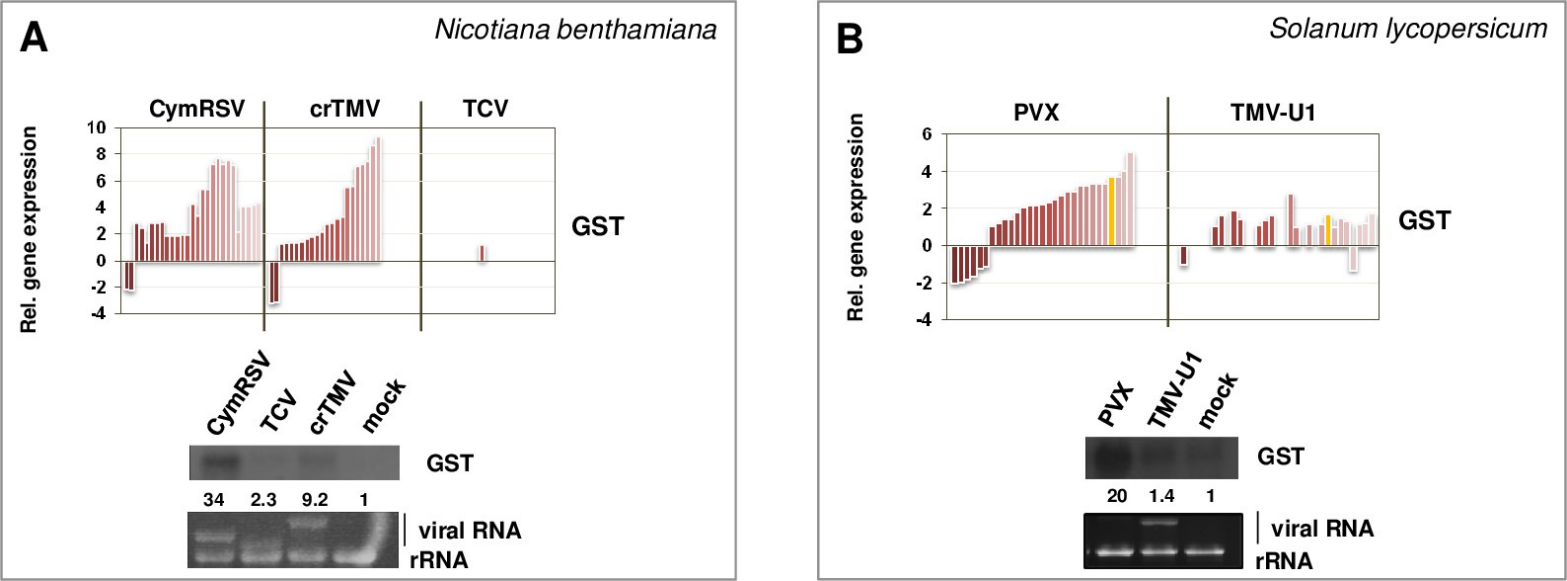
**Investigation of the gene expression changes of GST in virus-infected *N*. *benthamiana* (A) and *S*. *lycopersicum* (B) plants.** The column diagram shows log2-fold changes of probes or genes specific for GST obtained by microarray analysis (*N*. *benthamiana*) or by RNA-seq (*S*. *lycopersicum*) (upper panels). The lower panel shows Northern blot hybridization using radioactively labelled GST-specific probe. Fold-change values are shown below each band (quantifications were normalized to mock controls). The EtBr-stained gel served as a loading control. Yellow columns depict genes whose levels were validated by Northern blot hybridization. (The investigated *N*. *benthamiana* GST probe was absent on the microarray chip).

According to the microarray analysis in *N*. *benthamiana* and RNA-seq analysis in *S*. *lycopersicum* plants, the expression of tubulin, EFs and histones was mostly downregulated during acute virus infections (CymRSV, crTMV and PVX), whereas they were not altered in persistent virus infections (TCV and TMV-U1) (S5A and S5B Fig). Quantitative RT-PCR results of EF and histone in PVX- and TMV-U1-infected *S*. *lycopersicum* plants validated these findings, showing downregulation only during acute (PVX) infection (S5C Fig. Glutathione S transferases, pathogen-related proteins, systemic acquired resistance genes (SARs) and heat shock proteins (HSPs) are typical stress genes: the expression of GST-specific probes in microarray experiments showed up-regulation in CymRSV- and crTMV-infected plants, whereas it remained unaltered during TCV infection (Fig 4A upper panel). According to the RNA-seq results, the GST genes of *S*. *lycopersicum* were usually upregulated during virus infection, and these changes were more severe in PVX-infected plants (Fig 4B upper panel). These changes were validated by Northern blot analysis (Fig 4A and 4B lower panels).

We checked the relative expression of PR, SAR and HSP transcripts in our microarray and RNA-seq data (S6 Fig); all were upregulated only during acute (CymRSV, crTMV and PVX) infections, whereas their induction was absent or mild during persistent (TCV or TMV-U1) infections. Induction of the pathogen-related protein Q gene (PR-Q), pathogen-related protein 1 gene (PR1) and SAR in *N*. *benthamiana* and HSP20 in *S*. *lycopersicum* was also validated by Northern blot analysis (S6A and S6B Fig).

Protodermal factor 1 (PDF1) and lactate dehydrogenase (LDH) were among the DEPs whose levels were severely changed in acute infection in *N*. *benthamiana* plants but stayed unchanged during persistent infection (S7A and S7B Fig). LDH was shown to play a role in proline metabolism [31]. During stress conditions, fast degradation of proline happens as a result of the activation of proline dehydrogenase (ProDH) [32]. Although lactate is a substrate of LDH, it is also a competitive inhibitor of ProDH (S7A Fig, lower panel at right). The increase in LDH expression during virus infection enhances ProDH activity, allowing a fast response to stress conditions. PDF1 encodes a putative extracellular proline-rich protein exclusively expressed in the L1 layer of meristems and the protoderm of organ primordia. Its level was strongly downregulated in acute infection compared to mock- and TCV-infected leaves (S7B Fig right panel). In *S*. *lycopersicum*, changes in PDF1 and LDH expression showed the same trend. PDF1 was markedly upregulated during PVX and only slightly in TMV-U1 infection. LDH was downregulated during PVX infection and slightly upregulated during TMV-U1 infection (S7A and S7B Fig right panels).

The gene expression patterns analysed above show that specific transcriptome changes are induced during acute infections. These changes are not characteristic of persistent infections. Acute transcriptome changes, however, are consistently similar in the two investigated hosts.

### Photosynthesis-related, cell wall-remodelling and hormone-responsive transcripts are severely altered during acute infection

Although extensively studied, the molecular background of viral symptom development is still elusive. Symptom development in virus-infected plants reflects the sum of various molecular and physiological changes [5, 33]. Chlorosis, yellowing, leaf size reduction, malformation or senescence are ‘general’ symptoms caused by several plant viruses. These changes usually coincide with alterations in photosynthetic activity or molecular structure of the host chloroplast [34–39], but insights into molecular changes are still elusive.

**Fig 5:**
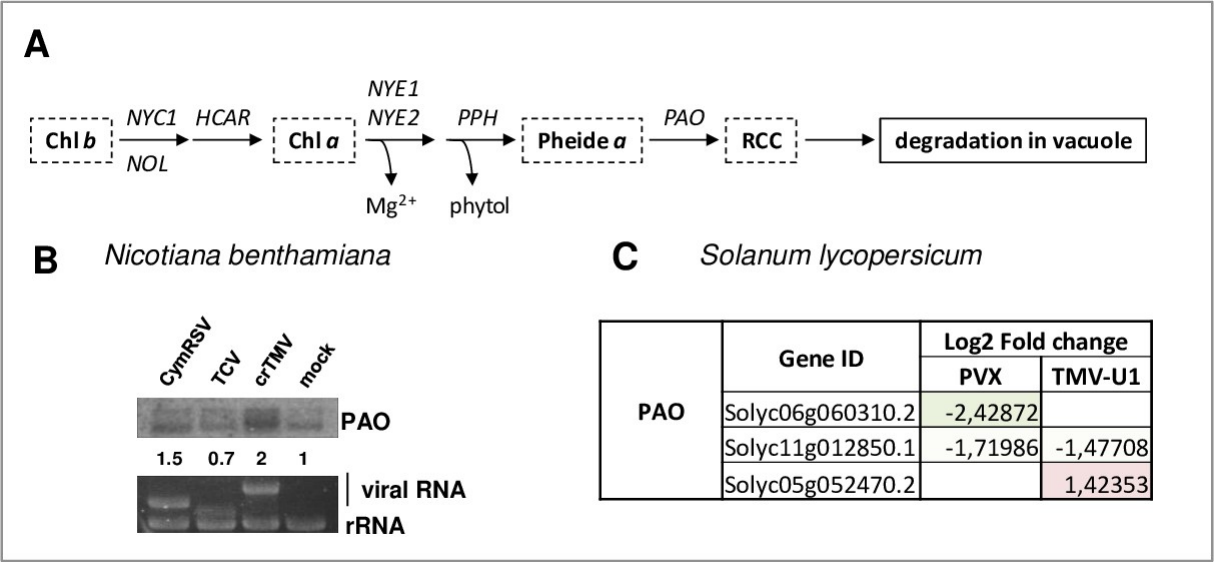
Investigation of the PAO gene expression changes in virus-infected plants. (A) Schematic diagram of the chlorophyll degradation pathway; (B) Northern blot hybridization using radioactively labelled PAO specific probe. Fold-change values are shown below each band (quantifications were normalized to mock control). EtBr-stained gel serves as a loading control. (C) The table shows gene expression changes of *S*. *lycopersicum* PAO transcripts. (listed in S13 Table with their characteristic parameters). (Chl b: chlorophyll b; NYC1: non-yellow coloring 1; NOL: nyc1-like HCAR: 7-hydroxymethyl Chl a reductase; Chl a: chlorophyll a; NYEs (NYE1, NYE2): non yellowing; PPH: pheophytinase; Pheide a: pheophorbide a; PAO: pheophorbide a oxygenase; RCC: red Chl catabolite).

Chlorosis and yellowing can be a result of chlorophyll (Chl) degradation, an early mark of leaf senescence. The key regulator of Chl degradation is pheophorbide a oxygenase (PAO), which catalyses the cleavage of the porphyrin ring of pheophorbide, resulting in a red Chl catabolite intermediate (RCC), which later degrades in the vacuole (Fig 5A). The level of PAO increases during biotic stresses [40, 41]. As on the microarray chip, we did not find a specific probe for *N*. *benthamiana* PAO, we showed by Northern blot analysis that its level was induced during acute (CymRSV and crTMV) infections (Fig 5B). By contrast to this observation, our RNA-seq results in *S*. *lycopersicum* showed three different PAO-like DEGs, whose expression slightly decreased in PVX and slightly altered in TMV-U1 infection (Fig 5C, S13 Table).

**Fig 6:**
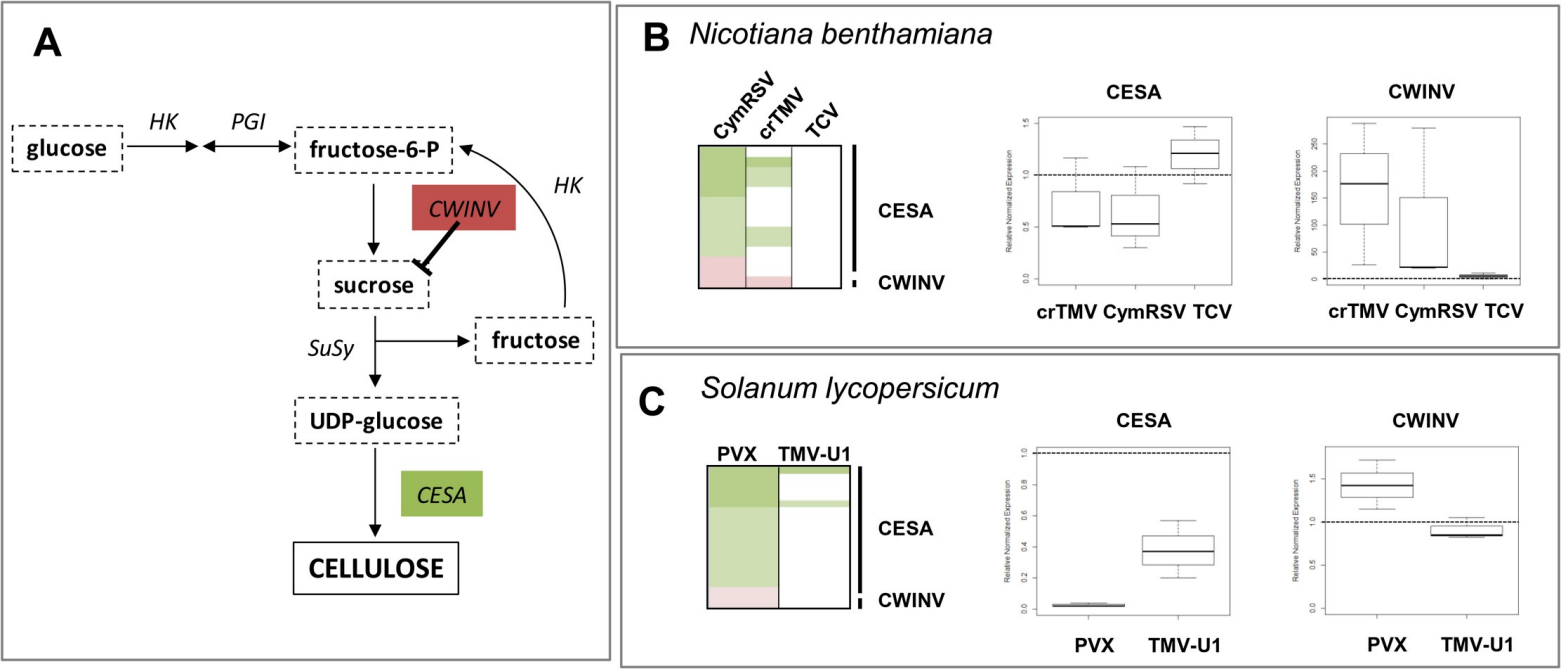
Investigation of the gene expression changes in cell wall metabolism. (A) Schematic diagram of cellulose synthesis pathway; (B, C) heat maps show gene expression changes of the investigated genes (left panels upregulation: red, downregulation: green). Whisker diagrams show delta-delta Ct results of qRT-PCR from 3 biological and technical replicates using ubiquitin as an internal control (middle and right panels). The DEPs and DEGs on the heat map are listed in S13 and S14 Tables with their characteristic parameters. (HK: hexokinase; PGI: phosphoglucose isomerase; CWINV: cell wall invertase; SuSy: sucrose synthase; CESA: cellulose synthase).

During acute infection, in addition to chlorosis and yellowing, slight stunting of both hosts was observed. When cell wall synthesis is impaired, the leaf size decreases, malformation of the leaf shape occurs, and the total growth of the plant is reduced. These changes can be observed in cell wall synthase (CESA) mutants or CESA-silenced plants [42]. By contrast, cell wall invertase (CWINV), which converts sucrose into fructose, is usually induced early during any defence [43] (Fig 6A left panel). With tomato yellow leaf curl virus, infected *S*. *lycopersicum* plants, especially when co-infected with tomato chlorosis virus, stunting was directly correlated to CESA8 down- and CWINV2 upregulation [34]. Investigation of the DEPs in *N*. *benthamiana* and the DEGs in *S*. *lycopersicum* that play a role in cell wall metabolism showed the same trend (Fig 6, S13 and S14 Tables); that is, the levels of the CESA8-type synthases were downregulated, whereas the invertases were upregulated. The findings were validated by qRT-PCR (Fig 6B and 6C).

According to our results, PAO, CESA and CWINV expression levels correlated very well with the type of infection, being more severe during acute infections in both hosts. These changes probably have a role in the development of the stunted phenotype during acute infections.

Different morphological abnormalities which are usually present in acute infection can also be the result of changes in hormone metabolism (reviewed recently in [5]). Auxins, cytokinins, gibberellic acid and brassinosteroids that regulate plant growth, development and elongation can have ambiguous effects during virus infection (reviewed by [44]). Although improper annotation did not allow us to map DEPs and DEGs of these pathways completely, after compiling gene expression changes of all probes and genes annotated to these pathways, these pathways were observed to be differentially regulated in acute infection, whereas changes were almost absent in persistent infection in both hosts (S15 and S16 Tables). Transcription factors regulated by other hormones, such as ethylene, abscisic acid, jasmonic acid and salicylic acid, can differentially regulate senescence-associated genes (SAGs), which finally lead to chlorophyll breakdown and leaf senescence [45, 46]. To summarize our transcriptomics results obtained by microarray and RNA-seq experiments, all hormone metabolism annotated changes were collected (S17 and S18 Tables) and visualized as heat maps of these pathways (S8 Fig). The overall or combined effects of hormone biosynthesis and regulation would finally lead to the development of leaf senescence. Changes at each level were more pronounced in the acute infections, which could explain the development of more severe symptoms during this type of infection.

### Physiological changes in virus-infected *N*. *benthamiana* and *S*. *lycopersicum* plants

**Fig 7:**
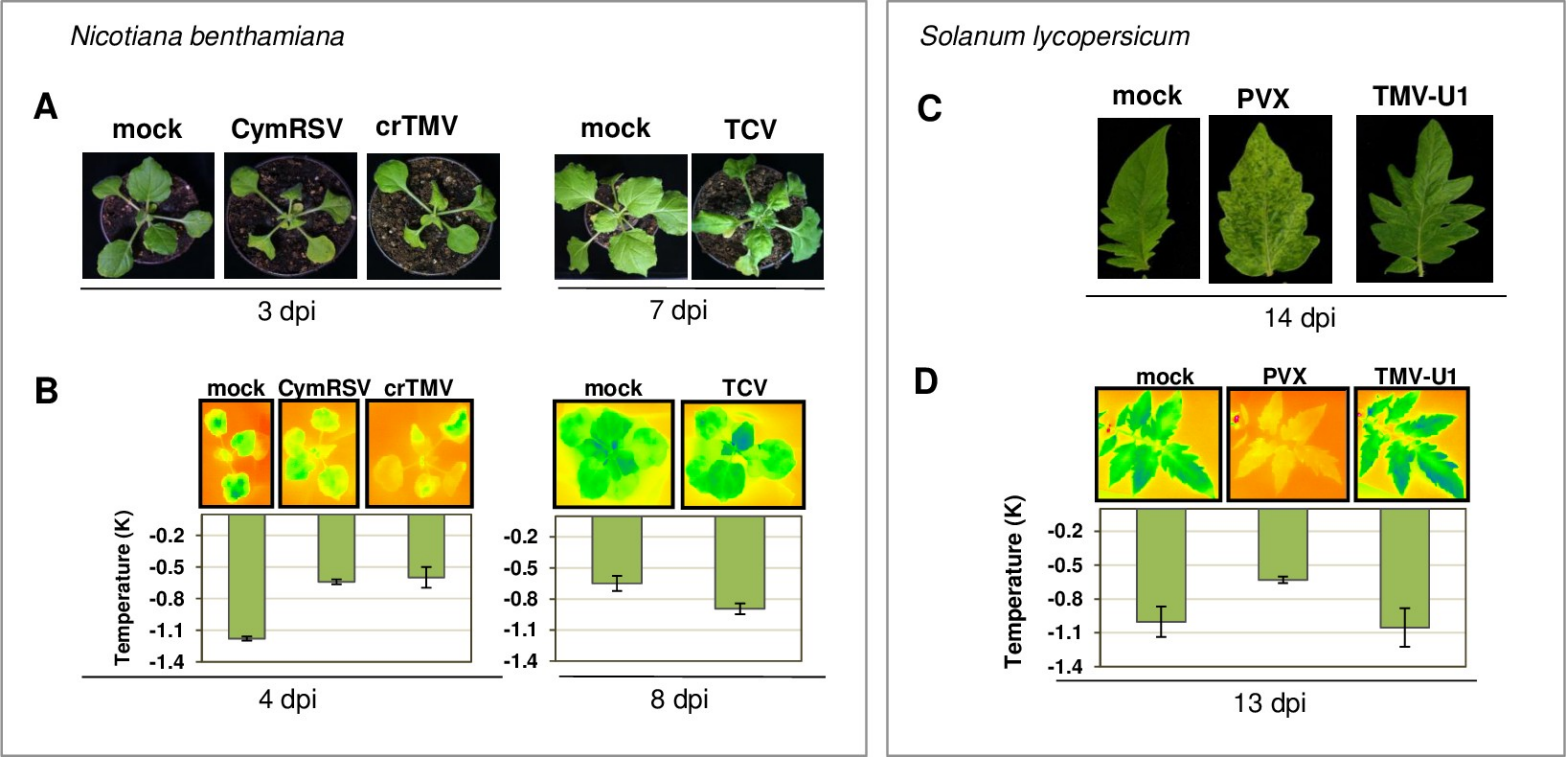
Symptoms and temperature changes in virus-infected *N*. *benthamiana* and *S*. *lycopersicum* plants. Photos in visible light (A) and thermographic images (B) of mock-inoculated and virus-infected *N*. *benthamiana* (A, B) and *S*. *lycopersicum* plants (C, D). Thermographic images show the difference between the ambient temperature and the leaf temperature of the virus-infected and control plant as a colour image and as a column diagram (in Kelvin, K).

**Fig 8:**
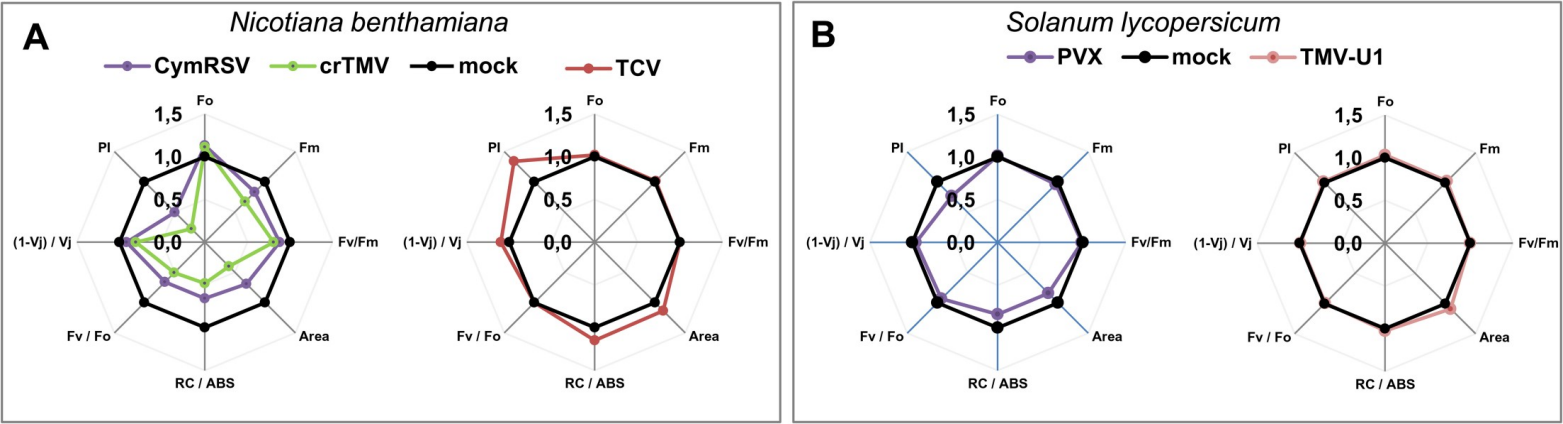
Chlorophyll fluorescence in virus-infected *N*. *benthamiana* and *S*. *lycopersicum* plants. Spider graphs show calculated chlorophyll fluorescence parameters of (A) *N*. *benthamiana* infected with CymRSV, crTMV (7 dpi) and TCV (11 dpi) and (B) *S*. *lycopersicum* infected with PVX and TMV (8 dpi). Parameters shown are initial (Fo) and maximal (Fm) fluorescence levels; the Fv/Fm and Fv/Fo (maximal PSII quantum yield) ratios; the area parameter, the dissipated energy flux per active reaction centre (RC/ABS); the (1-Vj)/ Vj parameter, where Vj = (F2ms–Fo)/Fv; and the performance index (PI) measured on the third/fourth young fully developed branches from the apical tip. The data refer to virus-infected plants (colour symbols) after normalization with respect to values obtained in the mock-inoculated plants (black symbols). Data are means of 3 independent biological plant replicates per treatment.

To check whether the molecular changes caused by the acute infection (vs persistent ones) are able to cause physiological alterations, we characterized the photosynthetic response of the host in the above host-virus interactions by the application of various physiological measurements (chlorophyll fluorescence, leaf temperature, area photosynthetic activity and calculated performance index (PI)) (Fig 7 and Fig 8).

Evaporative cooling of healthy and infected plants could be distinguished by using thermal imaging approaches. With better stomatal regulation, leaves of healthy plants transpire more water than is transpired by infected plants, which can be the effect of stomatal closure induced by the pathogen. Thermal images of acute and persistent virus infections were quantified by thresholding the evaporative cooled area relative to the temperature of the surrounding air (Fig 7). We could observe similar transpiration rates similar to those of the mock-infected plants during persistent (TCV- and TMV-U1 -infected *N*. *benthamiana* and *S*. *lycopersicum*, respectively) infections. By contrast, during acute (CymRSV, crTMV and PVX) infections the leaf temperature was found to be significantly higher (almost similar to that of ambient air), suggesting that acute viruses have a drastic negative impact on the evapotranspiration of their hosts.

The chlorophyll fluorescence fast kinetics approach also proved to be very effective in measuring physiological responses. Chlorophyll fluorescence parameters characterizing the electron transfer on both the acceptor and the donor sides of photosystem II drastically affected the performance index parameter, the maximal quantum yield for primary photochemistry and the quantum yield for electron transport [47, 48] in CymRSV- and crTMV-infected *N*. *benthamiana* and in PVX-infected *S*. *lycopersicum* plants. In TCV-infected *N*. *benthamiana* and TMV-U1-infected *S*. *lycopersicum* plants, the chlorophyll fluorescence parameters were the same or a little higher than in the control (Fig 8A and 8B).

Physiological data are consistent with molecular changes, and the mild alterations of gene expression during persistent infection do not alter the physiology of the leaves. During acute infection, the severe transcriptome changes may cause physiological alterations as observed even at a very early time-point of the infection.

### Expression of different regulators active in the nucleus showed the same pattern during different infections

Molecular and physiological changes in *N*. *benthamiana* and *S*. *lycopersicum* plants followed the same pattern in the different viral lifestyles. Severe changes occurred during acute infection but were absent or mild during persistent infection in both hosts, suggesting the presence of a universal regulatory pathway. *In vitro* transcription assays have proven that shut-off happens at the transcription level in the nucleus [22], where transcription factors regulate their targets. During acute infection, these types of DEPs in *N*. *benthamiana* were seriously altered. The expression of six of them was confirmed by Northern blot analysis, and their possible role is discussed in the Supplementary Material (S1 File and S9 Fig). In the nucleus of virus-infected plants, where transcription factors regulate their targets, the transcription factors themselves are often under the control of RNAi-based regulation executed by microRNAs (miRNA) and small interfering RNAs (siRNA) (reviewed by [5, 49, 50]). During transcriptional gene silencing (TGS) 24-nt-long small RNAs are loaded into Argonaute 4 protein (AGO4) to direct changes in DNA methylation and histone modification. The TGS may be modulated during pathogen attack [51]. When analysed, AGO4 was found to be differently regulated; its level was downregulated only in acute infection. AGO4 mRNA changes were also validated by Northern blot analysis in *N*. *benthamiana* (S10A Fig). Methyltransferases changed in a similar manner (S10B Fig).

### Persistent downregulation of housekeeping genes during acute infection is not a consequence of necrosis

**Fig 9:**
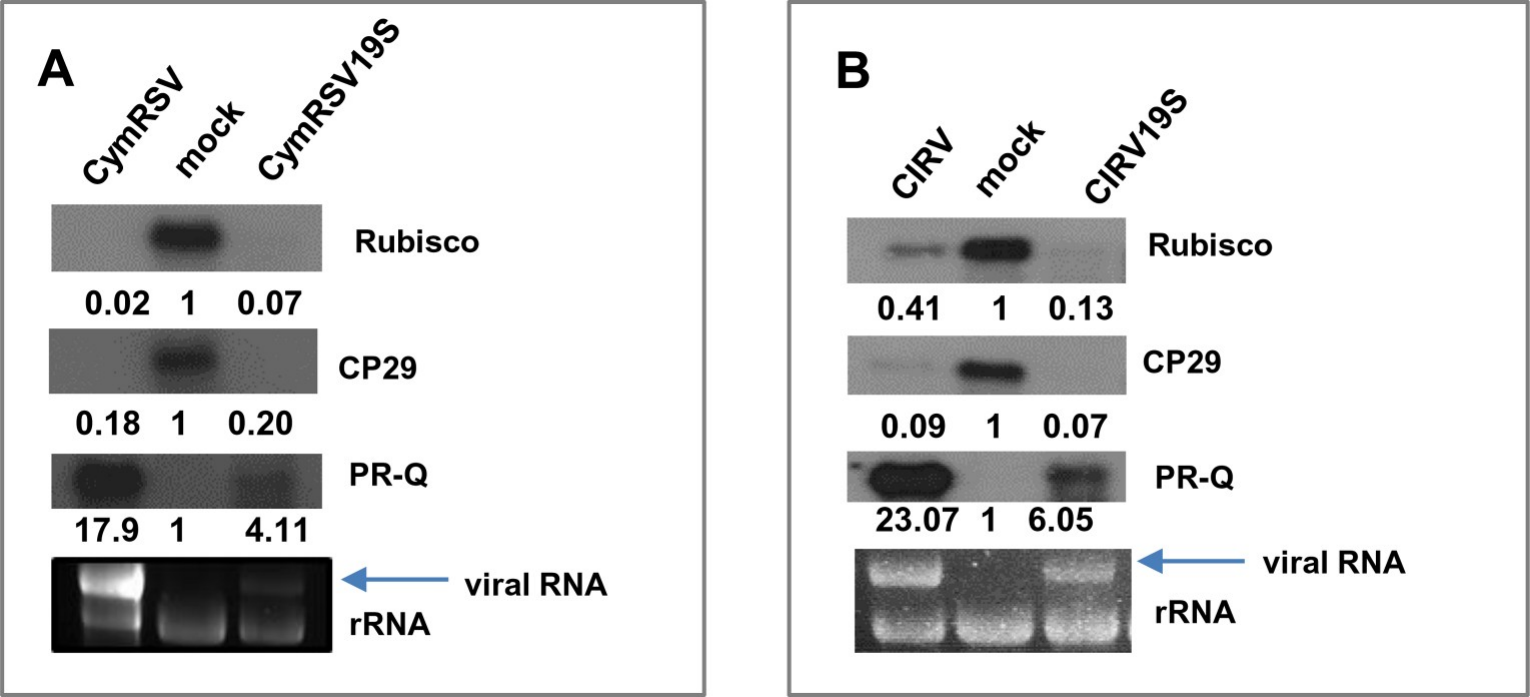
Northern blot analysis of the gene expression changes in *N*. *benthamiana* plants infected with wild-type and VSR mutant viruses at 15°C. *N*. *benthamiana* plants were infected with CymRSV or CymRSV19S (A) and CIRV or CIRV19S viruses (B). Accumulation of Rubisco, CP29 and PR-Q mRNA was investigated in systemically infected leaves at 12 dpi by Northern blot analysis. Fold-change values are shown below each band (quantifications were normalized to mock controls). EtBr-stained agarose gel served as a loading control.

As we have shown, acute infections have a profound effect on the gene expression pattern and physiology of the hosts. However, whether the molecular and physiological changes are the cause or the consequence of the early necrosis is not clear. Tombusviruses, such as CymRSV and CIRV, systemically infect *N*. *benthamiana* plants, and the infection ultimately culminates in the death of the host. These viruses encode a very efficient viral suppressor protein of RNA silencing (VSR), p19. p19 binds virus-derived small interfering RNA (vsiRNA) duplexes in a size-specific manner [52], consequently blocking their loading into RNA-induced silencing complexes and thus inhibiting the antiviral RNA silencing defence [53]. During infection with suppressor-deficient mutant viruses (CymRSV19S and CIRV19S), however, the antiviral system works efficiently, and the plants are able to recover from infection [54]. After a recovery period, an almost healthy phenotype develops (recovery phenotype). In recovery plants, due to the efficient antiviral silencing, the virus is present only at a low level. The efficiency of RNA silencing decreases at lower temperatures [55]. Under low-temperature conditions, suppressor-deficient viruses can also accumulate to a high level. It was also shown that CymRSV19S or CIRV19S viruses do not induce necrosis even at low temperatures [56]. To exclude the possibility that downregulation of housekeeping genes is a consequence of early necrosis, we analysed housekeeping gene expression patterns in response to CymRSV19S- and CIRV19S-infection at 15°C. Samples from newly developed, systemically infected leaves were collected at 12 dpi, and the expression levels of two housekeeping genes (Rubisco, CP29) and a stress gene (PR-Q) were investigated by Northern blot analysis (Fig 9).

The Rubisco and CP29 levels were downregulated and the expression of PR-Q was induced irrespective of the presence of the VSR. Similarly, as we showed earlier two other housekeeping transcript (GAPDH and tubulin) levels were reduced during CymRSV19S infection [22]. These findings suggest that the shut-off of housekeeping genes is not a consequence of the upcoming necrosis or the presence of VSR.

## Conclusions

In our work, we investigated genome-wide gene expression changes and physiological alterations during acute and persistent infections in two different hosts. Persistent shut-off of housekeeping- and photosynthesis-related transcripts, induction of stress-related transcripts and alterations of cell wall remodelling enzyme transcripts were exclusively caused by viruses that had an acute lifestyle strategy, but not in the case of persistent viruses. Gene expression changes on the global scale (microarray and RNA-seq data) were validated by an alternative method (Northern blot analysis or qRT-PCR) for representative transcripts of each class. Acute virus-specific changes were very similar in the two host plants (*N*. *benthamiana* and *S*. *lycopersicum*) and in the case of different viruses (Tombusviruses, Tobamoviruses and Potexviruses), thereby suggesting that general mechanisms are involved. Two methods were used to serve as a cross-reference in our study, validating the reliability of the results and strengthening our conclusions.

During infection, viruses harness the host machinery to replicate themselves. During this process, the host endomembrane system, RNA transport, transcription and translation machinery are hijacked by the virus (deployed from the host). This shortage of host enzymatic processes may be directly or indirectly responsible for acute virus-caused gene expression changes and subsequent physiological alterations. However, the extremely high virus level during persistent infection in the investigated host-virus systems (TCV-infected *N*. *benthamiana* and TMV-U1-infected *S*. *lycopersicum* plants) contradict this hypothesis and suggest that extreme overuse of the host transcriptional and translational system is probably not the primary cause of severe host gene alteration during acute infections.

We show that the housekeeping gene shut-off is not the consequence of the upcoming necrosis or the presence of RNA silencing suppressor protein. Previously it was proposed that interference of the silencing suppressor with endogenous silencing pathways might contribute to viral symptom development [57, 58]. This possibility has since been questioned [59] because the p19 suppressor is not able to bind endogenous small RNAs in a virus-infected environment. We propose, therefore, that the severe transcriptome and physiological changes caused by the acute viruses but not alterations of the miRNA-guided developmental pathway are the primary contributors of viral symptoms. Our finding that key regulators of TGS (AGO4) were altered during acute infection implies that the altered TGS-mediated pathway could cause broad-range changes in host gene regulation. Mapping 24-nt small RNA-mediated methylation changes of the host in this type of infection could answer this question in the future. Moreover, as has been shown for AGO1, translational regulation (which cannot be detected by either microarray or RNA-seq experiments) can further influence this complex pattern.

While investigating the mechanism of shut-off in virus-infected mammalian cells, Gilbertson and colleagues recently showed that cytoplasmic degradation of mRNAs can negatively regulate their initial transcription rate in the nucleus [60]. During mRNA degradation, RNA-binding proteins are released and shuttle back to the nucleus, where they block early stages of transcription. During virus infection, the translation machinery is redirected to produce viral proteins, which may affect both host RNA stability and protein translation, leading to the induction of gene expression changes as observed during acute infections. Beside cell-autonomous impacts, viruses may alter plant metabolism on tissue or organism level (water and nutrient transport), which would affect cell-autonomous regulatory pathways. Testing how these regulatory mechanisms operate in virus-infected plants could further contribute to our understanding of host-virus interactions and development of viral symptoms.

We used two different high-throughput methods in our experiments: microarray hybridization and RNA sequencing to detect changes in the gene expression pattern of the host in compatible virus infections. Changes in expression levels were higher in the case of DEPs compared to DEGs, which could be a result of different detection methods. The ratio of downregulated genes in microarray analysis was higher compared to that in upregulated ones, which could be a result of the presence of overrepresented probes. This trend is a warning signal that interpretation of the most extensively changed gene ontology categories cannot be precisely defined based on the microarray data.

Severe downregulation of genes that play a role in photosynthetic and other housekeeping processes has a marked effect on plant physiology. Thermal imaging and variable chlorophyll fluorescence transients to follow leaf temperature and photosynthetic activity, respectively, could measure these effects. According to our results, physiological changes associated with the extensive shut-off of host genes in acute infections make it possible to differentiate the latter from persistent infections, even before visible symptoms appear. Remote sensing methods are able to monitor these physiological changes [61–63]; however, the development and use of these new technologies must be based on detailed knowledge of both the changes in gene expression and the resulting physiological changes in virus-infected plants. Consequently, our data could be utilized in the future by remote sensing techniques in precision agriculture, helping crop management by predicting possible infection risks.

High-throughput sequencing methods lead to the discovery of more and more viruses each day, but determining the actual importance (disease-causing potential) of these newly discovered viruses is very difficult. Characterizing gene expression patterns of housekeeping genes and identifying the type of viral infection based on their expression levels could be used in risk assessments (Table 1).

**Table 1:** Summary of basic differences between acute and persistent virus infections which could be used to predict type of the infection.

Gene-expression changes							
	Rubisco	GAPDH	CPs	GST	Physiological parameters		
Acute	decrease			increase		leaf temperature	Chl fluorescence parameters
Persistent	no change			no change	Acute	increase	decrease
					Persistent	no change	no change
	LDH	PAO	CWINV				
Acute	increase						
Persistent	no change						
	PDF	CESA					
Acute	decrease						
Persistent	no change						

Coexistence of a virus and its host in a persistent virus infection without visible symptoms could be beneficial for the virus and the host at an evolutional scale. Interestingly, the persistently infecting TCV caused improved physiological attributes (e.g., Performance index increased by 1.4-fold, Fig 8A), suggesting that TCV may be a mutualist rather than an agent of disease. Although we gained deep insight into the regulation of gene expression in various viral infections, how viruses can avoid or minimize the host reaction during persistent infections to reach a ‘peaceful’ coexistence with their host is a very exciting question that needs to be further investigated.

## Supporting information

S1 Fig Sampling strategy of virus-infected *N. benthamiana* plants. (A) Photo of infected *N*. *benthamiana* plants at the time of microarray sampling, together with the dpi-s for the different types of experiments. (B) Samples from different biological replicates used for microarray were checked for the endogenous GAPDH level by Northern blot analysis. The photo of the EtBr-stained agarose gel served as a loading control. The presence of TCV, CymRSV and crTMV were validated according to their RNA size. Numbers show relative expression of the investigated gene, where 1 is the expression level of the gene in mock-inoculated sample.
(TIF)

S2 Fig Sampling strategy of virus-infected *S. lycopersicum* plants. (A) Experimental design for the different types of experiments, with sample collection time in dpi being indicated with photos of virus-infected and mock-inoculated plants. (B) Northern blot analysis of individual plant extracts in the first experiment probed hybridized with radioactively labelled virus-specific probe. Samples of virus-infected Kecskeméti jubileum cultivar was also tested for the endogenous Rubisco (C) level by Northern blot analysis after being hybridized with a radioactively labelled gene-specific probe. EtBr-stained gel served as a loading control. Numbers show relative expression of the investigated gene, where 1 is the expression level of the gene in the mock-inoculated sample.
(TIF)

S3 Fig **Functional distribution of all differentially expressed probes or genes (up and downregulated) probes or genes in all virus infected (A) *N*. *benthamiana* and (B) *S*. *lycopersicum* plants**. Functions were grouped according to their Bin codes. All DEPs and DEGs are listed with their Bin codes and other characteristic parameters in the S1 and S2 Tables.
(TIF)

S4 Fig **Comparison of the DEPs/DEGs that play a role in (A) photosynthesis and (B) stress responses.**Heat map of DEPs in virus-infected *N*. *benthamiana* and DEGs in virus infected *S*. *lycopersicum* plants were prepared using green for downregulated and red for upregulated genes. The intensity of the colour correlates with the severity of the changes. White shows changes that are smaller than 2-fold. A list of probes and genes whose levels are indicated on the heat map, along with their characteristic parameters, is detailed in the S3 and S4 Tables (for photosynthesis) and in the S5 and S6 Tables (for stress responses).
(TIF)

S5 Fig **Investigation of the gene expression changes of tubulin, elongation factors (Efs) and histones in virus-infected (A) *N*. *benthamiana* and (B) *S*. *lycopersicum* plants**. The column diagrams show log2-fold changes of probes or genes specific for the investigated gene resulting from (A) *N*. *benthamiana* microarray analysis or from (B) *S*. *lycopersicum* RNAseq. (C) Validation of gene expression changes of *S*. *lycopersicum* EF and histone coding gene by quantitative RT-PCR. Box plot shows the relative gene expression calculated from delta-delta Ct values in 3 biological and 3 technical replicates for each gene in the control and the PVX- or TMV-infected plant, using ubiquitin as an internal control. The yellow shows gene expression changes of (A) probes specific for tubulin and EF, whose level was investigated in our previous work (Havelda et al, Plant Journal 2008), or (B) the level for a specific gene that was validated by qRT-PCR.
(TIF)

S6 Fig **Gene expression changes of stress-related genes in (A) *N*. *benthamiana* and (B) *S*. *lycopersicum* plants.** The column diagrams show log2-fold changes of probes or genes specific for the investigated gene resulted from (A) *N*. *benthamiana* microarray analysis or from (B) *S*. *lycopersicum* RNAseq. Yellow shows gene expression changes of (A) probes or (B) genes whose level was validated by Northern blot analysis. In Northern blot experiments, the membrane was hybridized with a radioactively labelled gene-specific probe. EtBr-staining served as a loading control. Numbers show relative expression of the investigated gene, where 1 is the expression level of the gene in the mock-inoculated sample.
(TIF)

S7 Fig Investigation of the gene expression changes of enzymatic pathways in virus-infected plants. Gene expression changes of (A) lactate dehydrogenase, (B) protodermal factor1, Schematic diagrams (A left panel) show role; tables show gene expression changes of the investigated gene, with red showing upregulation and green showing downregulation. Northern blot hybridizations used radioactively labelled gene-specific probes. EtBr staining served as the loading control.
(TIF)

S8 Fig Summary of the DEPs and DEGs that play role in hormone regulation identified during virus infection in *N. benthamiana* and *S. lycopersicum* plants. Boxes show the heat map results of the DEPs or DEGs specific for the genes that play a role in hormone metabolism. The intensity of the colour correlates with the magnitude of the change. Green shows downregulation, whereas red shows upregulation. The list of probes and genes whose levels are indicated on the heat map is detailed in the S17 Table (*N*. *benthamiana*) and S18 Table (*S*. *lycopersicum*), together with their characteristic parameters.
(TIF)

S9 Fig Investigation of the gene expression changes of regulator factors in virus-infected *N. benthamiana* and S. lycopersicum plants. (A) Panels show log2-fold changes of probes or genes specific for the investigated gene obtained by microarray analysis (*N*. *benthamiana*) or by RNA-seq (*S*. *lycopersicum*). (B) Gene expression changes of regulator genes were investigated by Northern blot hybridization using radioactively labelled probes specific for endogenous genes. EtBr-stained agarose gel served as a loading control.
(TIF)

S10 Fig Investigation of the gene expression changes of AGO4 and methyltransferases in virus-infected plants. Gene expression changes as log2-fold changes of probes or genes specific for (A) AGO4 and (B) methyltransferases are shown on right panels. (A) Gene expression changes of AGO4 were investigated by Northern blot hybridization using AGO4-specific radioactively labelled probe. EtBr-stained agarose gel served as a loading control. (B) Gene expression changes of methyltransferases are shown also as a column diagram.
(TIF)

S1 Table Gene expression changes in virus-infected *N. benthamiana* plants identified by microarray hybridization. The list of probes that showed differential expression in any of the virus-infected plants. Log2-fold change values, along with their corresponding *p* values, are indicated if higher than 2 and less than 0.05 in CymRSV-, crTMV-, and TCV-infected *N*. *benthamiana*. Description and GO annotation of the probe and its function according to Bin categories are also indicated.
(XLSX)

S2 Table Gene expression changes in virus-infected *S. lycopersicum* plants identified by RNA seq. List of genes that showed differential expression in any of the virus-infected plants. Log2-fold change values, along with their corresponding *p* values, are indicated if higher than 2 and less than 0.05 in PVX- and TMV-U1-infected *S*. *lycopersicum*. Description and GO annotation of the gene and its function according to Bin categories are also indicated.
(XLSX)

S3 Table Summary of the DEPs that play a role in photosynthetic processes in virus-infected *N. benthamiana* plants shown in S4A Fig. Log2-fold change values, along with their corresponding *p* values, are indicated if higher than 2 and less than 0.05 in CymRSV-, crTMV-, and TCV-infected *N*. *benthamiana*. Description and GO annotation of the probe and its function according to Bin categories are also indicated. The corresponding gene from S. lycopersicum is also shown together with its log2-fold and *p* value if it was identified as a DEG in the PVX- or TMV-infected tomato.
(XLSX)

S4 Table Summary of DEGs that play a role in photosynthetic processes in virus-infected *S. lycopersicum* plants shown in S4A Fig. Log2-fold change values, along with their corresponding *p* values, are indicated if higher than 2 and less than 0.05 in PVX- and TMV-infected *S*. *lycopersicum*. Description and GO annotation of the probe and its function according to Bin categories are also indicated.
(XLSX)

S5 Table Summary of DEGs that play a role in stress responses in virus-infected *N. benthamiana* plants shown in S4B Fig. Log2-fold change values, along with their corresponding *p* value, are indicated if higher than 2 and less than 0.05 in CymRSV-, crTMV- and TCV-infected *N*. *benthamiana*. Description and GO annotation of the probe and its function according to Bin categories are also indicated.
(XLSX)

S6 Table Summary of DEGs that play a role in stress responses in virus-infected *S. lycopersicum* plants shown in S4B Fig. Log2-fold change values, along with their corresponding *p* values, are indicated if higher than 2 and less than 0.05 in PVX- and TMV-infected *S*. *lycopersicum*. Description and GO annotation of the probe and its function according to Bin categories are also indicated.
(XLSX)

S7 Table The top 20 probes leading to downregulation in crTMV-, CymRSV- and TCV- infected *N. benthamiana* plants. Log2-fold change values, along with their corresponding *p* values, are indicated if higher than 2 and less than 0.05 Description and GO annotation of the probe and its function according to Bin categories are also indicated.
(XLSX)

S8 Table The top 20 probes leading to upregulation in crTMV-, CymRSV- and TCV- infected *N. benthamiana* plants. Log2-fold change values, along with their corresponding *p* values, are indicated if higher than 2 and less than 0.05 Description and GO annotation of the probe and its function according to Bin categories are also indicated.
(XLSX)

S9 Table The top 20 downregulated genes in PVX- and TMV-infected *S. lycopersicum* plants. Log2-fold change values, along with their corresponding *p* values, are indicated if higher than 2 and less than 0.05 Description and GO annotation of the probe and its function according to Bin categories are also indicated.
(XLSX)

S10 Table The top 20 upregulated genes in PVX- and TMV-infected *S. lycopersicum* plants. Log2-fold change values, along with their corresponding *p* values, are indicated if higher than 2 and less than 0.05 Description and GO annotation of the probe and its function according to Bin categories are also indicated.
(XLSX)

S11 Table List of primers used to amplify parts of the indicated endogenous gene from *N. benthamiana*. (XLSX)

S12 Table List of primers used to amplify parts of the indicated endogenous gene from *S. lycopersicum*. (XLSX)

S13 Table Summary of DEGs that play a role in chlorophyll degradation and cell wall metabolism in virus-infected *S. lycopersicum* plants shown in Fig 7 and Fig 8. Log2-fold change values, along with their corresponding *p* values, are indicated if higher than 2 and less than 0.05 in PVX- and TMV-infected *S*. *lycopersicum*. Description and GO annotation of the probe and its function according to Bin categories are also indicated.
(XLSX)

S14 Table Summary of DEGs that play a role in cell wall metabolism in virus-infected *N. benthamiana* plants shown in Fig 8. Log2-fold change values, along with their corresponding *p* value, are indicated if higher than 2 and less than 0.05 in CymRSV-, crTMV- and TCV- infected *N*. *benthamiana*. Description and GO annotation of the probe and its function according to Bin categories are also indicated.
(XLSX)

S15 Table Summary of DEPs that play a role in auxin, brassinosteroid, cytokinin and gibberellin metabolism in virus-infected *N. benthamiana* plants. Log2-fold change values, along with their corresponding *p* values, are indicated if higher than 2 and less than 0.05 in CymRSV-, crTMV-, and TCV-infected *N*. *benthamiana*. The description and GO annotation of the probe and its function according to Bin categories are also indicated. The corresponding gene from S. lycopersicum is also shown, along with its log2-fold and *p* value, if it was identified as a DEG in PVX- or TMV-infected tomato.
(XLSX)

S16 Table Summary of DEGs that play a role in auxin, brassinosteroid, cytokinin and gibberellin metabolism in virus-infected *S. lycopersicum* plants. Log2-fold change values, along with their corresponding *p* values, are indicated if higher than 2 and less than 0.05 in PVX-and TMV-infected *S*. *lycopersicum*. Description and GO annotation of the probe and its function according to Bin categories are also indicated.
(XLSX)

S17 Table Summary of DEPs that play a role in hormone metabolism in virus-infected *N. benthamiana* plants summarized in S7 Fig. Log2-fold change values, along with their corresponding *p* values, are indicated if higher than 2 and less than 0.05 in CymRSV-, crTMV- and TCV-infected *N*. *benthamiana*. Description and GO annotation of the probe and its function according to Bin categories are also indicated.
(XLSX)

S18 Table Summary of DEGs that play a role in hormone metabolism in virus-infected *S. lycopersicum* plants summarized on S7 Fig. Log2-fold change values together with their corresponding *p* value are indicated if higher than 2 and less than 0.05 in PVX- and TMV-infected S. lycopersicum. Description and GO annotation of the probe and its function according to Bin categories are also indicated.
(XLSX)

S19 Table List of primers used to amplify parts of the indicated viral genomes. PCR product amplified with them were used as a template for radioactively labelled virus-specific probes.
(XLSX)

S1 File Supporting gene expression results of possible regulators showing altered expression during acute infection. (DOCX)
