# Supplementary figures and images for: Differential gene expression and physiological changes during acute or persistent plant virus interactions may contribute to viral symptom differences
Source: PLoS One. 2019 May 3;14(5):e0216618. doi: 10.1371/journal.pone.0216618 (PMC6499435; doi:10.1371/journal.pone.0216618)

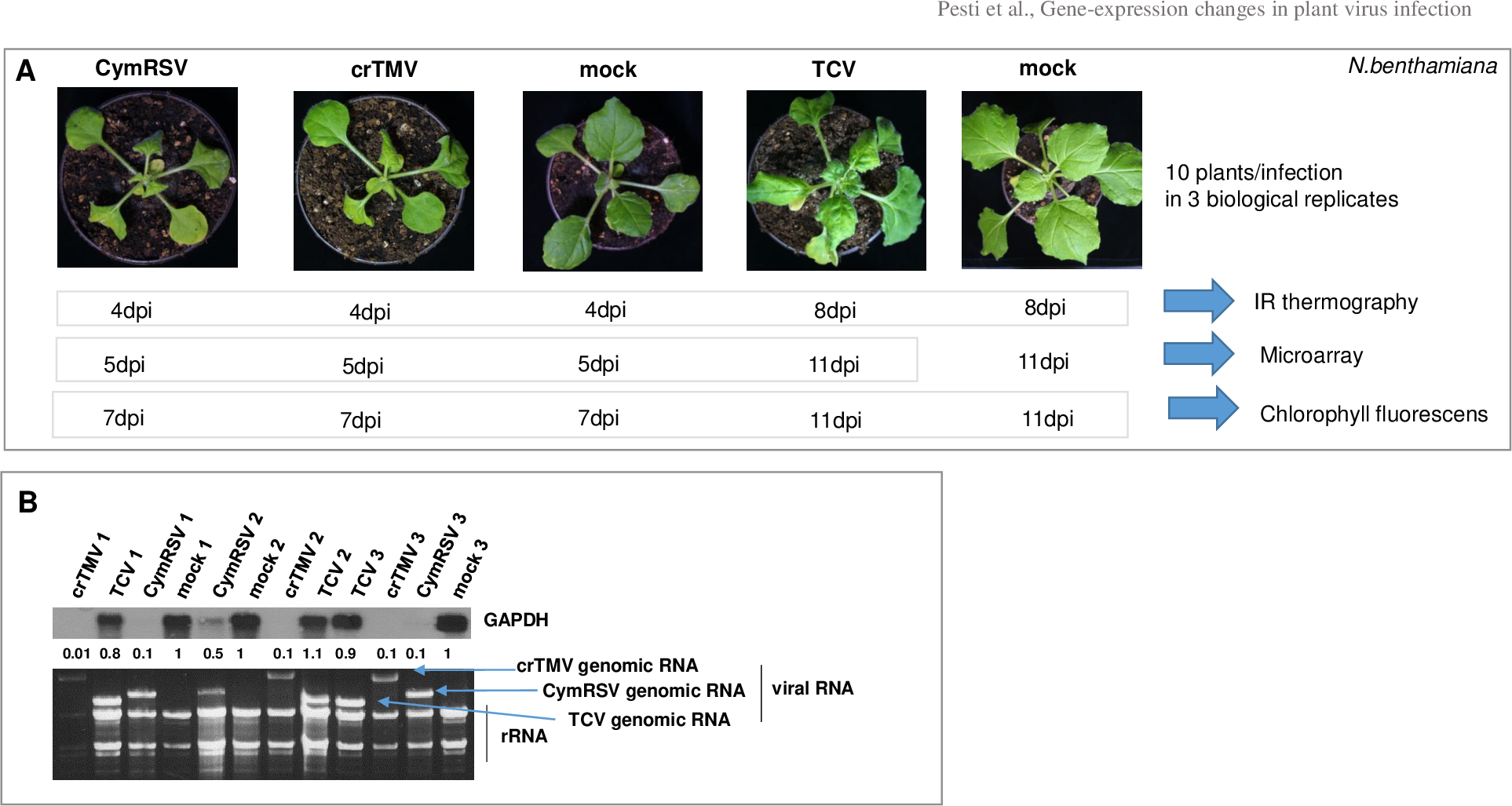

Supplement: S1 Fig — (A) Photo of infected N. benthamiana plants at the time of microarray sampling, together with the dpi-s for the different types of experiments. (B) Samples from different biological replicates used for microarray were checked for the endogenous GAPDH level by Northern blot analysis. The photo of the EtBr-stained agarose gel served as a loading control. The presence of TCV, CymRSV and crTMV were validated according to their RNA size. Numbers show relative expression of the investigated gene, where 1 is the expression level of the gene in mock-inoculated sample. (TIF) [file pone.0216618.s001.tif]

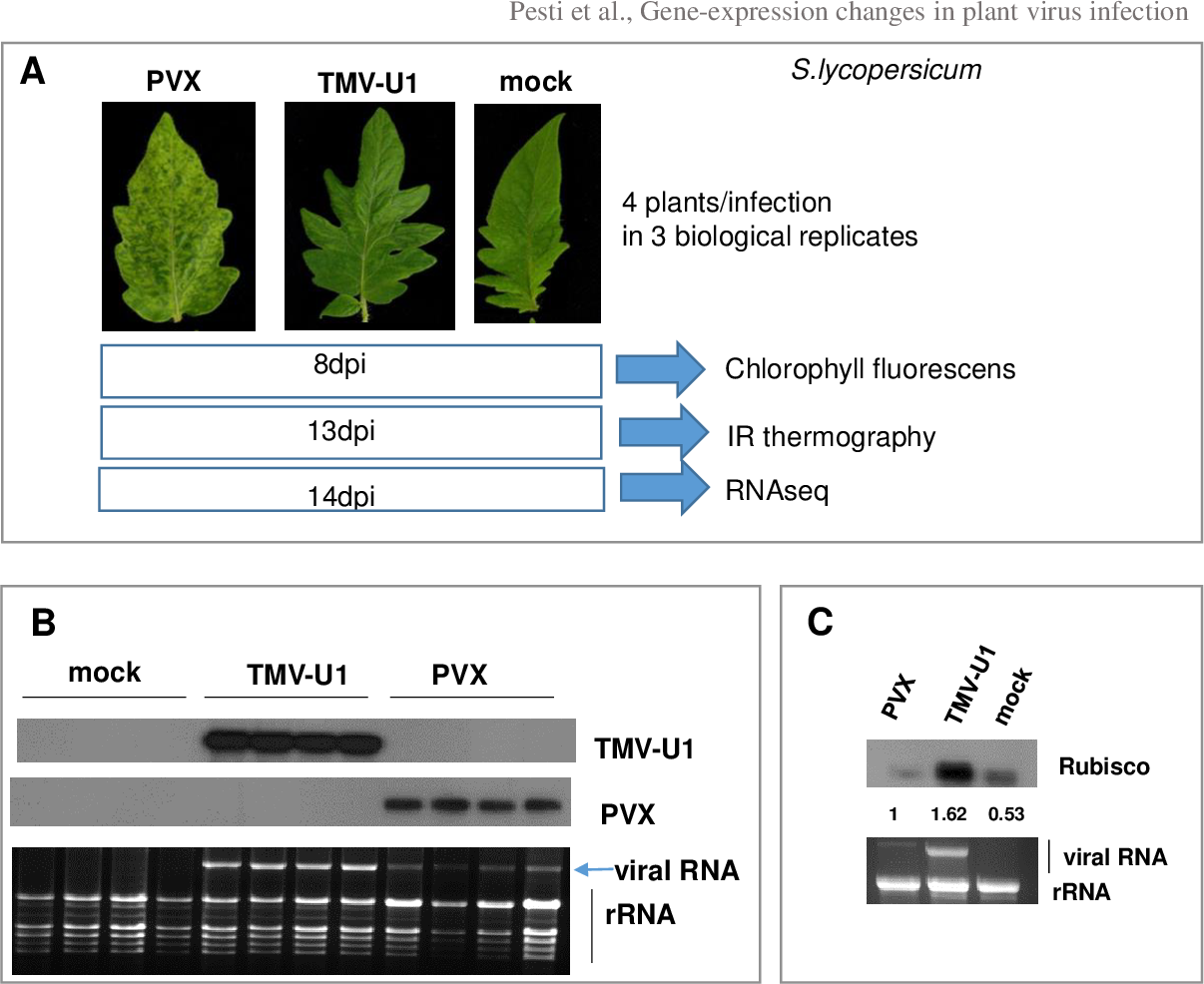

Supplement: S2 Fig — (A) Experimental design for the different types of experiments, with sample collection time in dpi being indicated with photos of virus-infected and mock-inoculated plants. (B) Northern blot analysis of individual plant extracts in the first experiment probed hybridized with radioactively labelled virus-specific probe. Samples of virus-infected Kecskeméti jubileum cultivar was also tested for the endogenous Rubisco (C) level by Northern blot analysis after being hybridized with a radioactively labelled gene-specific probe. EtBr-stained gel served as a loading control. Numbers show relative expression of the investigated gene, where 1 is the expression level of the gene in the mock-inoculated sample. (TIF) [file pone.0216618.s002.tif]

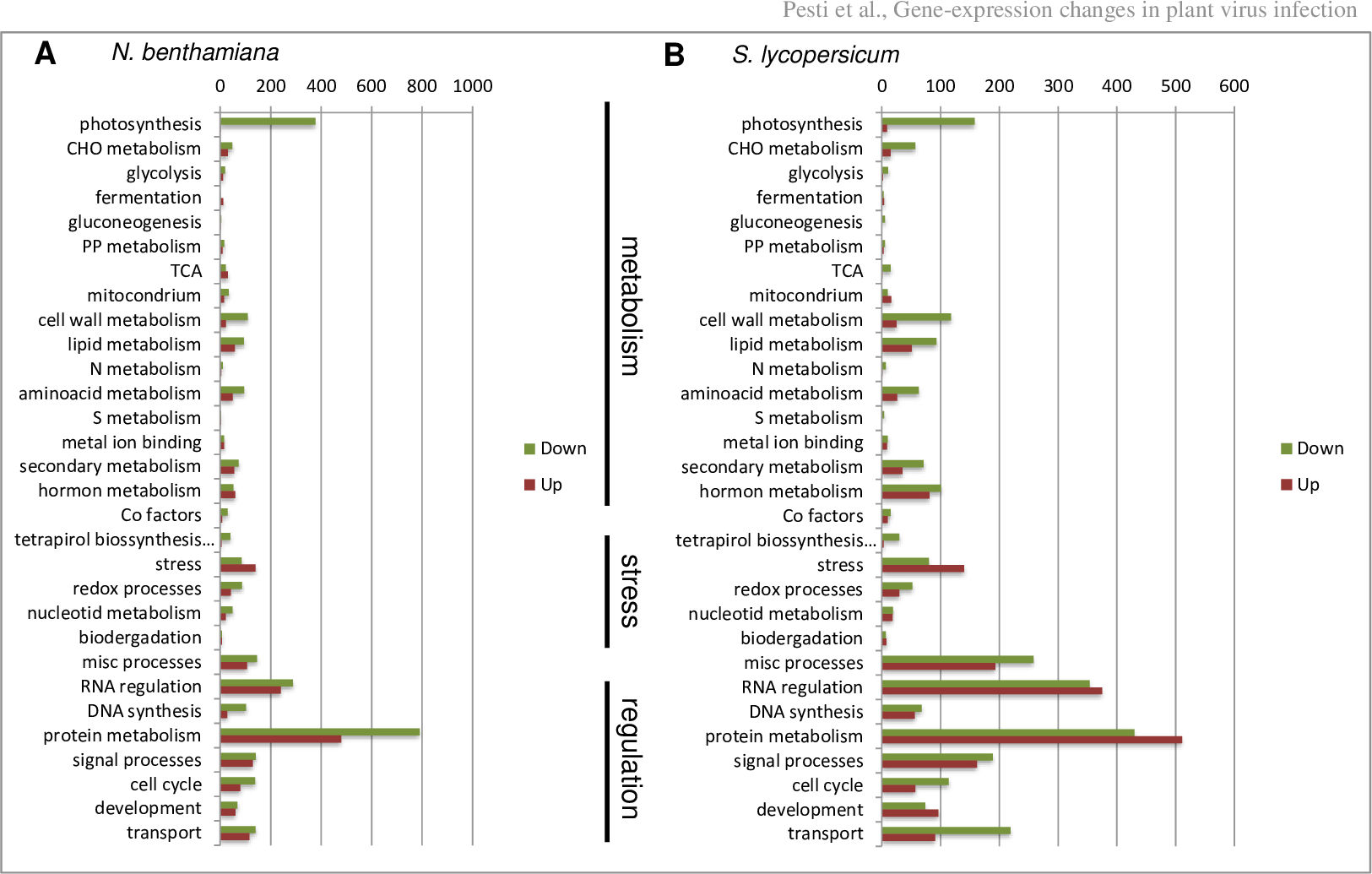

Supplement: S3 Fig — Functional distribution of all differentially expressed probes or genes (up and downregulated) probes or genes in all virus infected (A) N. benthamiana and (B) S. lycopersicum plants. Functions were grouped according to their Bin codes. All DEPs and DEGs are listed with their Bin codes and other characteristic parameters in the S1 and S2 Tables. (TIF) [file pone.0216618.s003.tif]

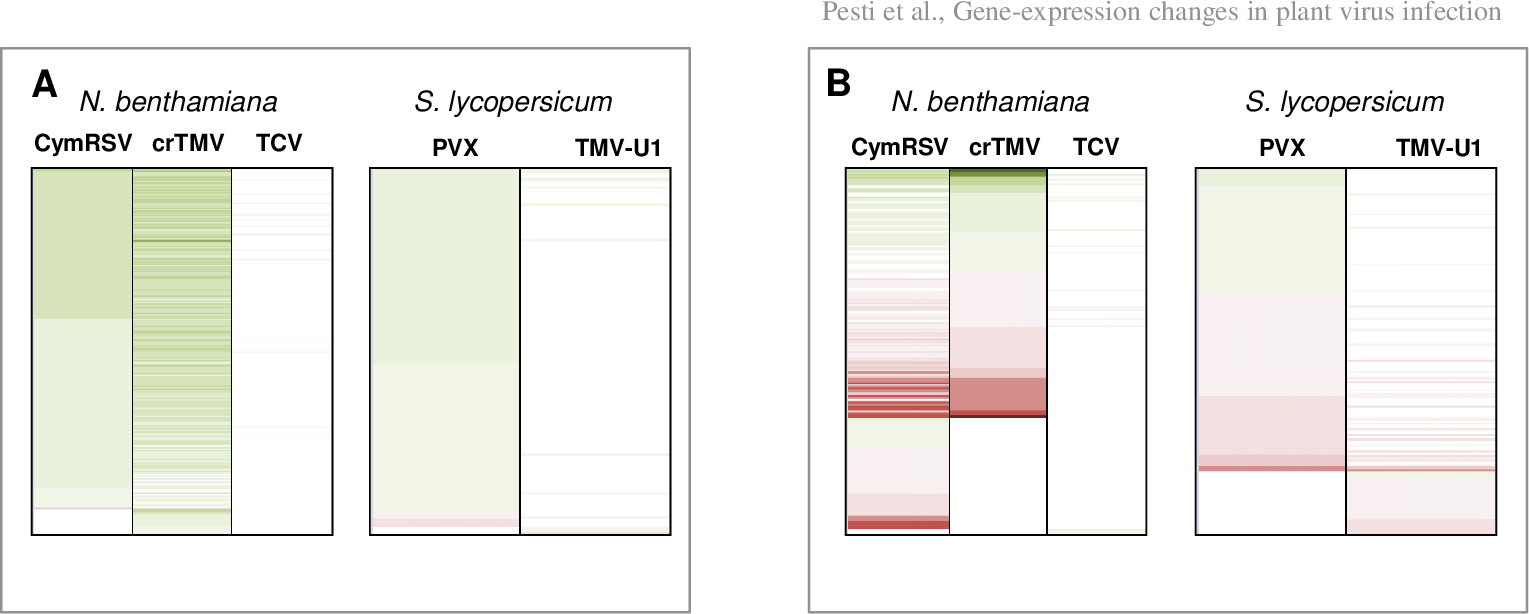

Supplement: S4 Fig — Comparison of the DEPs/DEGs that play a role in (A) photosynthesis and (B) stress responses.Heat map of DEPs in virus-infected N. benthamiana and DEGs in virus infected S. lycopersicum plants were prepared using green for downregulated and red for upregulated genes. The intensity of the colour correlates with the severity of the changes. White shows changes that are smaller than 2-fold. A list of probes and genes whose levels are indicated on the heat map, along with their characteristic parameters, is detailed in the S3 and S4 Tables (for photosynthesis) and in the S5 and S6 Tables (for stress responses). (TIF) [file pone.0216618.s004.tif]

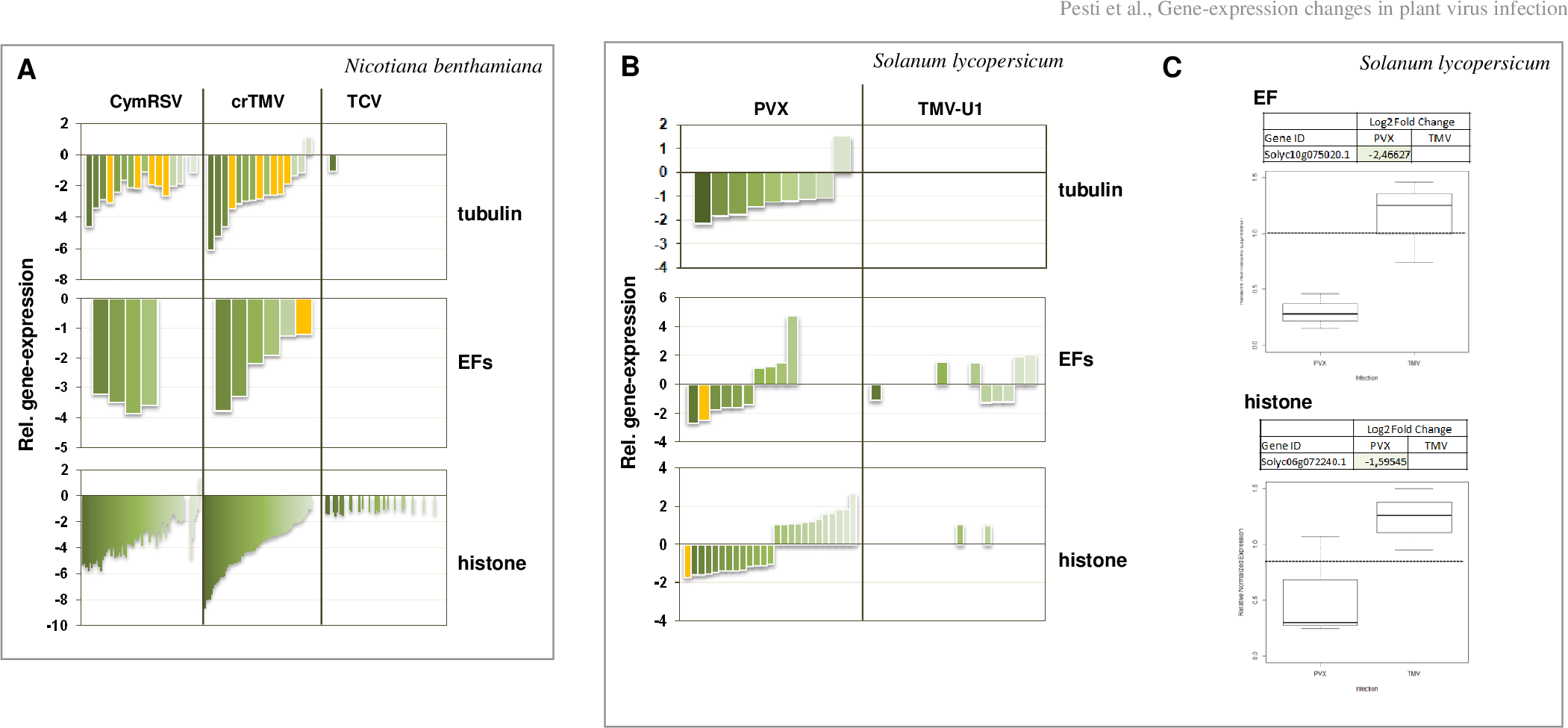

Supplement: S5 Fig — Investigation of the gene expression changes of tubulin, elongation factors (Efs) and histones in virus-infected (A) N. benthamiana and (B) S. lycopersicum plants. The column diagrams show log2-fold changes of probes or genes specific for the investigated gene resulting from (A) N. benthamiana microarray analysis or from (B) S. lycopersicum RNAseq. (C) Validation of gene expression changes of S. lycopersicum EF and histone coding gene by quantitative RT-PCR. Box plot shows the relative gene expression calculated from delta-delta Ct values in 3 biological and 3 technical replicates for each gene in the control and the PVX- or TMV-infected plant, using ubiquitin as an internal control. The yellow shows gene expression changes of (A) probes specific for tubulin and EF, whose level was investigated in our previous work (Havelda et al, Plant Journal 2008), or (B) the level for a specific gene that was validated by qRT-PCR. (TIF) [file pone.0216618.s005.tif]

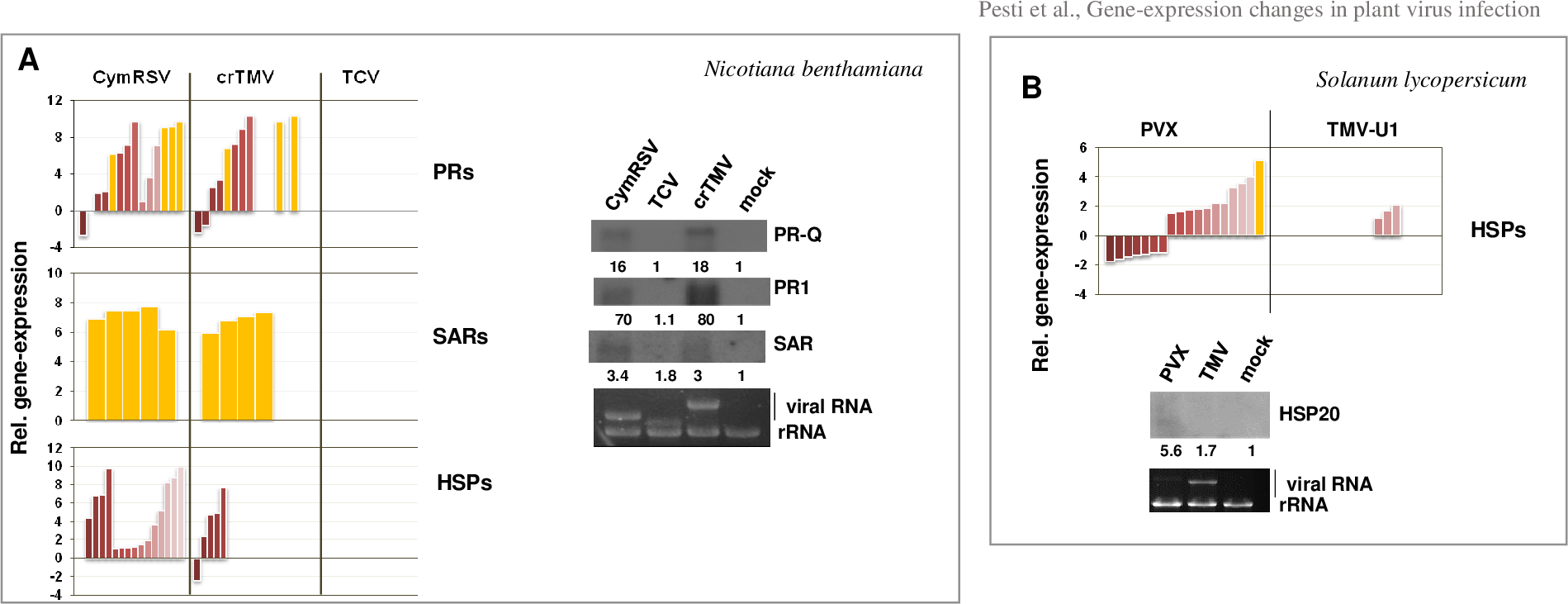

Supplement: S6 Fig — Gene expression changes of stress-related genes in (A) N. benthamiana and (B) S. lycopersicum plants. The column diagrams show log2-fold changes of probes or genes specific for the investigated gene resulted from (A) N. benthamiana microarray analysis or from (B) S. lycopersicum RNAseq. Yellow shows gene expression changes of (A) probes or (B) genes whose level was validated by Northern blot analysis. In Northern blot experiments, the membrane was hybridized with a radioactively labelled gene-specific probe. EtBr-staining served as a loading control. Numbers show relative expression of the investigated gene, where 1 is the expression level of the gene in the mock-inoculated sample. (TIF) [file pone.0216618.s006.tif]

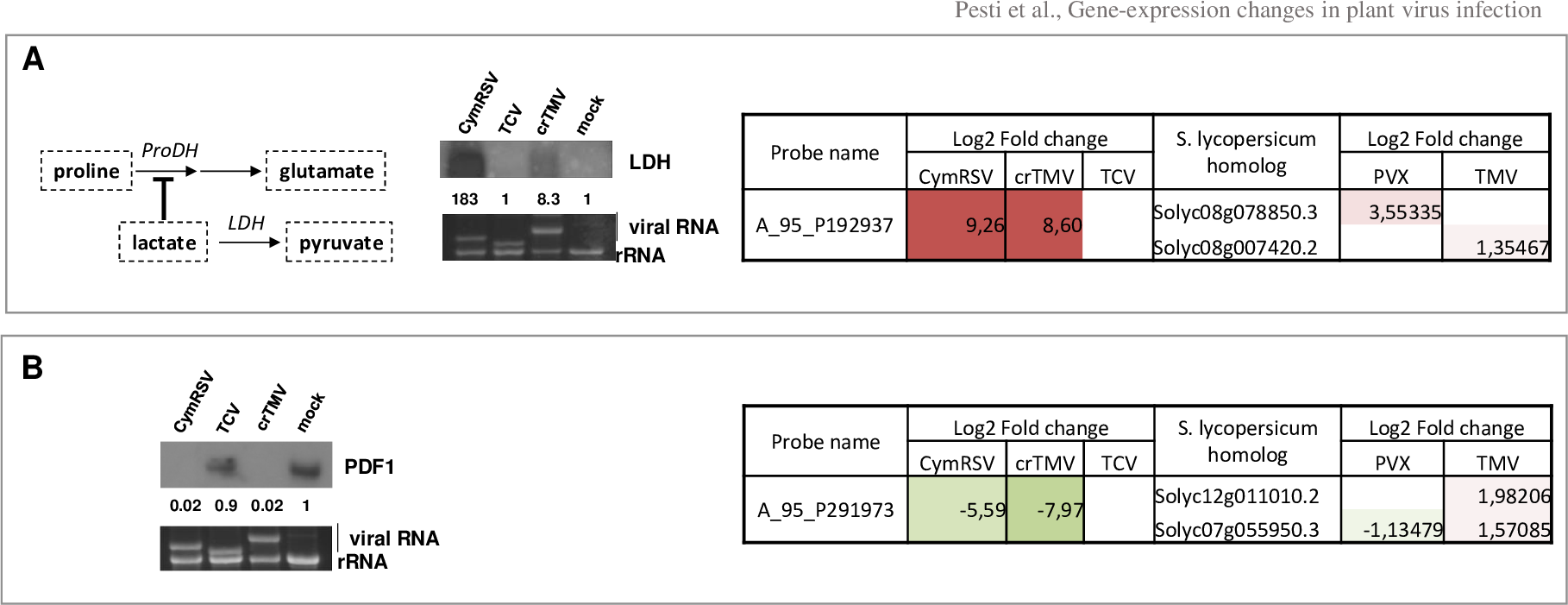

Supplement: S7 Fig — Gene expression changes of (A) lactate dehydrogenase, (B) protodermal factor1, Schematic diagrams (A left panel) show role; tables show gene expression changes of the investigated gene, with red showing upregulation and green showing downregulation. Northern blot hybridizations used radioactively labelled gene-specific probes. EtBr staining served as the loading control. (TIF) [file pone.0216618.s007.tif]

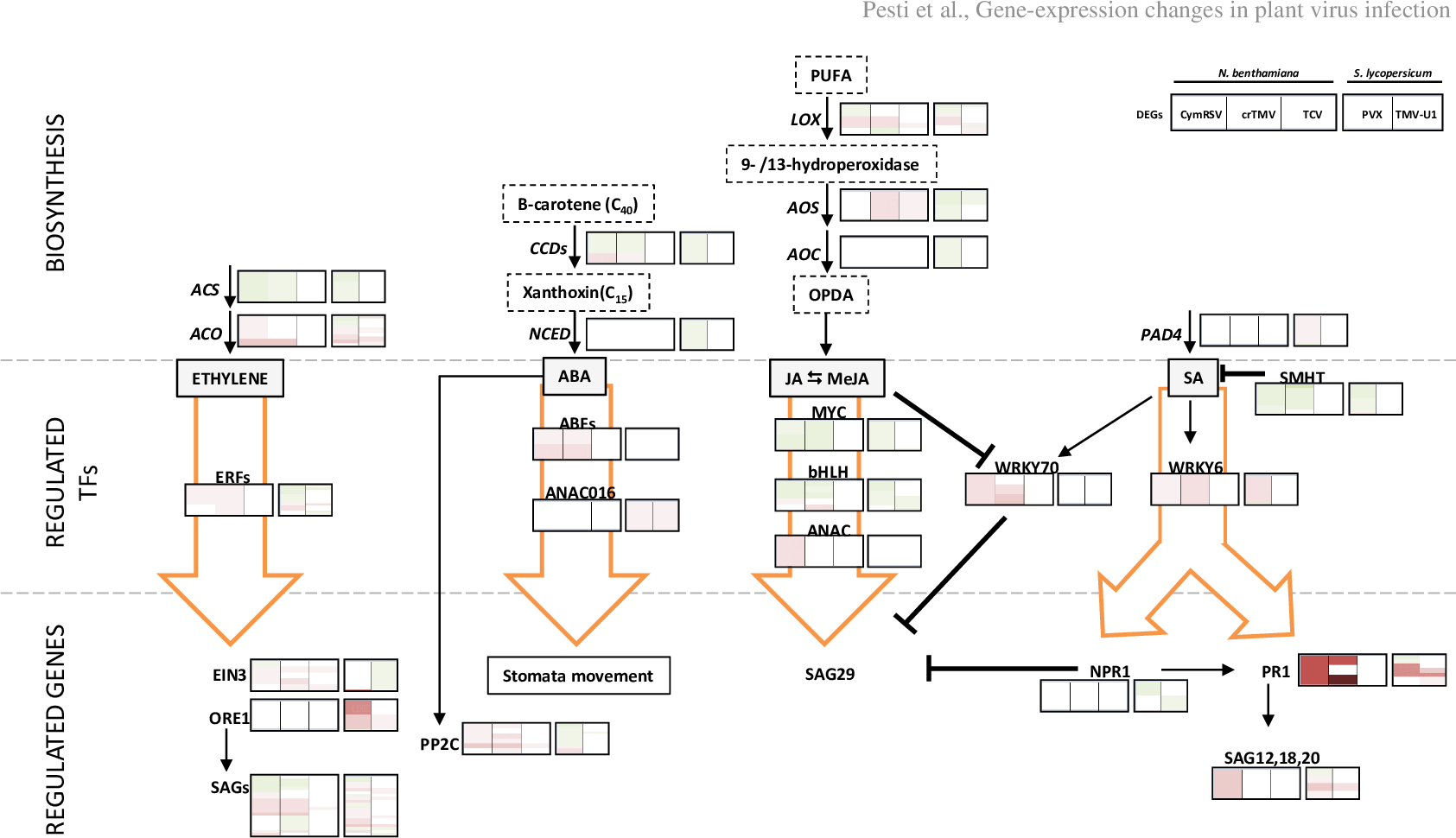

Supplement: S8 Fig — Boxes show the heat map results of the DEPs or DEGs specific for the genes that play a role in hormone metabolism. The intensity of the colour correlates with the magnitude of the change. Green shows downregulation, whereas red shows upregulation. The list of probes and genes whose levels are indicated on the heat map is detailed in the S17 Table (N. benthamiana) and S18 Table (S. lycopersicum), together with their characteristic parameters. (TIF) [file pone.0216618.s008.tif]

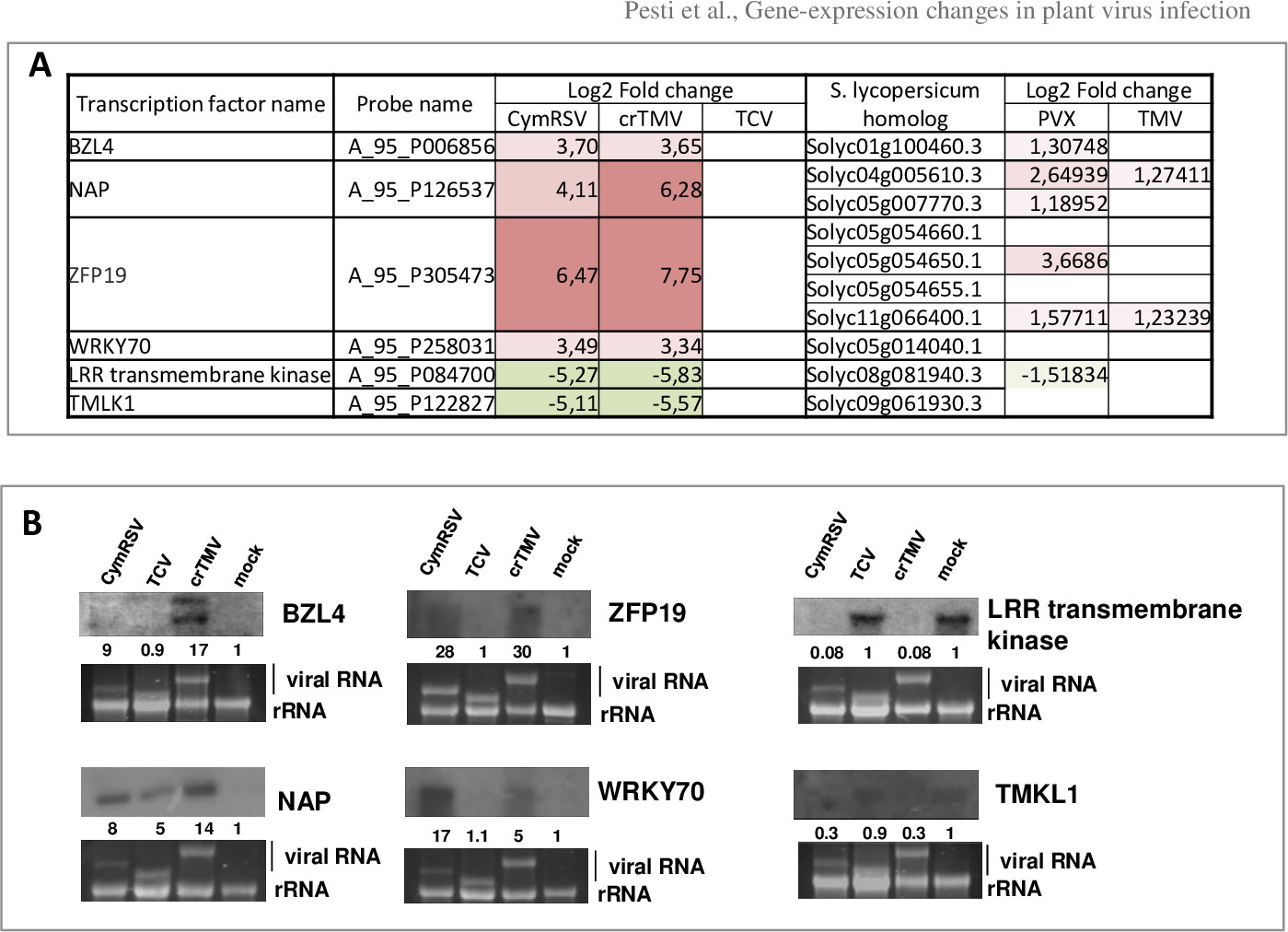

Supplement: S9 Fig — (A) Panels show log2-fold changes of probes or genes specific for the investigated gene obtained by microarray analysis (N. benthamiana) or by RNA-seq (S. lycopersicum). (B) Gene expression changes of regulator genes were investigated by Northern blot hybridization using radioactively labelled probes specific for endogenous genes. EtBr-stained agarose gel served as a loading control. (TIF) [file pone.0216618.s009.tif]

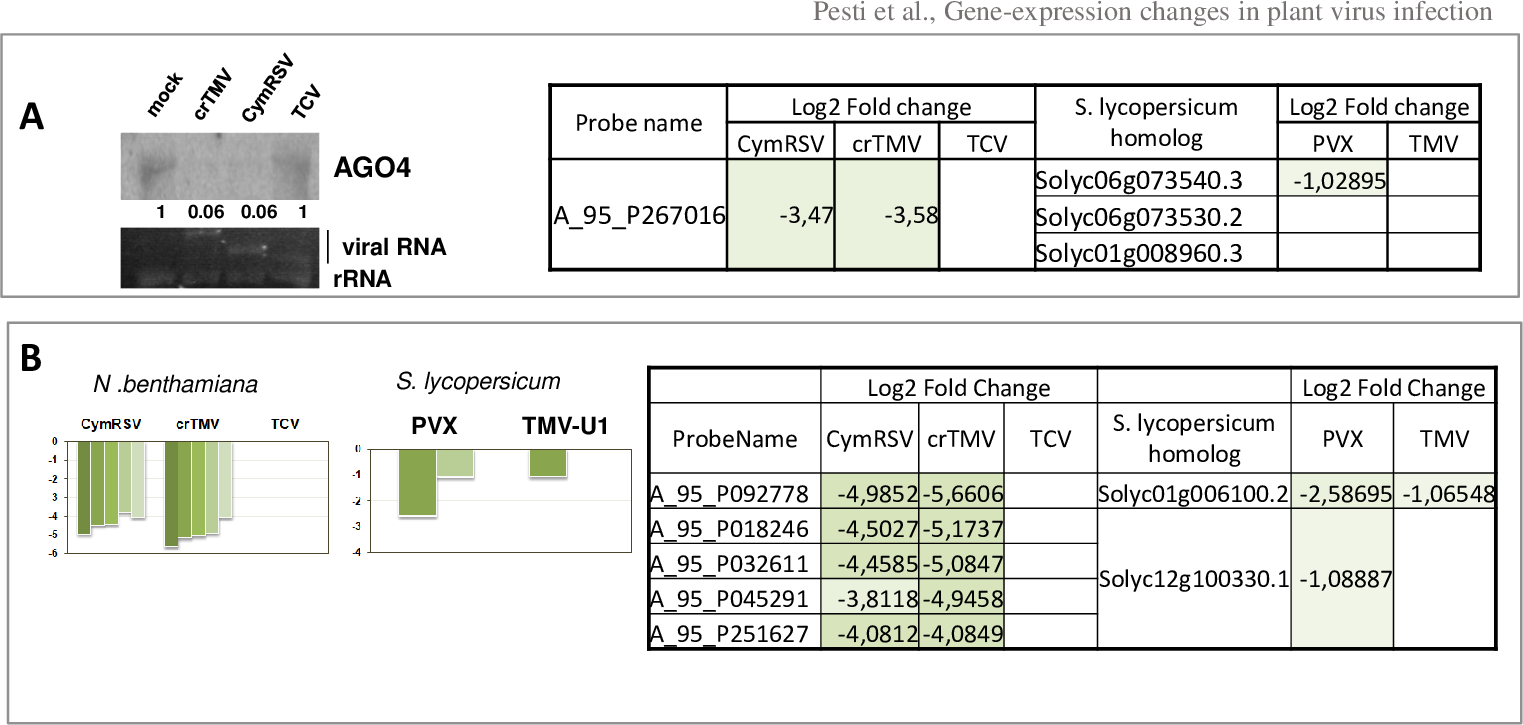

Supplement: S10 Fig — Gene expression changes as log2-fold changes of probes or genes specific for (A) AGO4 and (B) methyltransferases are shown on right panels. (A) Gene expression changes of AGO4 were investigated by Northern blot hybridization using AGO4-specific radioactively labelled probe. EtBr-stained agarose gel served as a loading control. (B) Gene expression changes of methyltransferases are shown also as a column diagram. (TIF) [file pone.0216618.s010.tif]
