# Supplementary material for: Differential gene expression and physiological changes during acute or persistent plant virus interactions may contribute to viral symptom differences
Source: PLoS One. 2019 May 3;14(5):e0216618. doi: 10.1371/journal.pone.0216618 (PMC6499435; doi:10.1371/journal.pone.0216618)
Supplement: S1 File — (DOCX) [file pone.0216618.s030.docx]

## S1 Text: Supporting results on gene expression of possible regulators showing altered expression during acute infection

The transcription levels of basic leucine zipper (BZL)-4, NAP (nucleosome assembly protein) like, zinc finger (ZF) P11 and WRKY70 transcription factors were markedly induced, whereas the levels of a leucin rich repeat (LRR) transmembrane kinase and a transmembrane kinase like (TMKL)-1 were reduced during acute infection (Supplementary Figure 9). BZL-4 is a member of the BZIP transcription factor family, with no information available about its role in biotic stresses. However, its overexpression in tobacco results stunted vegetative growth, shortened internodes, and smaller flowers and reduced amount of pollen ([1](#_ENREF_1)). Moreover, ectopic overexpression of its possible orthologues from *Antirrhinum majus* repressed transcription of the chlorophyll a/b (CAB) binding protein ([2](#_ENREF_2)). In line with this, plants with increased BZL-4 level, i.e. CymRSV- and crTMV-infected tobacco and PVX-infected tomato showed reduced growth and the levels of CABs were seriously downregulated, suggesting that this BZL transcription factor is regulated during biotic stresses. NAC-like transcription factors are well studied and shown to play an important role in leaf senescence and to be regulated by the ethylene insensitive (EIN)-2 pathway ([3](#_ENREF_3)). A NAP-like probe on the microarray chip was annotated as a homologue of the AtNAP (ANAC019-like) transcription factor, which is expressed in floral primordia and upregulated by APETALA 3 (AP3) and PISTILLATA (PI) ([4](#_ENREF_4)). Overexpression of AtNAP resulted in premature senescence ([5](#_ENREF_5)) and its increased level was connected to early senescence events ([6](#_ENREF_6)). Expression of its *N. benthamiana* and *S. lycopersicum* homologues was induced in acute infections, showing that in these infections early senescence had been started. Zinc finger (ZF) transcription factors play diverse roles under stress conditions, even during biotic stresses. AtZAT11 (At2g37430) expression was induced in response to the flagellin22 elicitor peptide during biotic stress in *Arabidopsis thaliana* ([7](#_ENREF_7)), whereas the level of PtiZFP1, a ZF11 homologue in poplar, was induced during SA and JA treatment and during rust infection ([8](#_ENREF_8)). In our experiments increased expression of probe P305473, annotated as a zinc finger transcription factor ZF11homologue was detected in acute infection in both hosts, suggesting that the regulatory pathway including these transcription factors is involved during acute virus infection. WRKY transcription factors are involved in the regulation of plant defense responses. WRKY70 itself has been shown to be induced by SA and repressed by JA, it interacts with NPR1, and through its activity represses defense-connected JA-responsive genes during biotic stress ([9](#_ENREF_9)). According to the latest results it is also involved in brassinosteroid-regulated plant growth ([10](#_ENREF_10)).The level of WRKY70 was induced during CymRSV, crTMV infection in *N. benthamiana* and during PVX infection in *S. lycopersicum*, suggesting its important role not only during bacterial and fungal, but also viral infection. LRR transmembrane kinase was annotated as a probable inactive kinase, but its *A. thaliana* homologue (At4g23740) was identified as activated (phosphorylated) during flagellin and xylanase treatment mimicking bacterial and fungal infection ([11](#_ENREF_11)). Expression of this LRR transmembrane kinase was decreased during CymRSV and crTMV infection in tobacco and in PVX-infected tomato (validated by Northern blot analysis in *N.benthamiana*), suggesting that it is active and may play a role in early signaling in virus infection. Another kinase, TMKL1 was also identified in our microarray experiments to be downregulated only during acute infection, which was validated by Northern blot analysis. This DEP represents a *N. benthamiana* homologue of an Arabidopsis transmembrane kinase, which has an unusual kinase-like domain ([12](#_ENREF_12)), but no known function. Expression of its tomato homologue showed no change during virus infection, thus its clear role during virus infection is not confirmed.

**References:**

1. Tim I, Anne S, Stefan B, Thomas Z, Thorsten H, Anne Gh, et al. Homo- and heterodimers of tobacco bZIP proteins counteract as positive or negative regulators of transcription during pollen development. The Plant Journal. 2010;63(1):155-66.

2. Strathmann A, Kuhlmann M, Heinekamp T, Dröge-Laser W. BZI-1 specifically heterodimerises with the tobacco bZIP transcription factors BZI-2, BZI-3/TBZF and BZI-4, and is functionally involved in flower development. The Plant Journal. 2001;28(4):397-408.

3. Kim HJ, Hong SH, Kim YW, Lee IH, Jun JH, Phee B-K, et al. Gene regulatory cascade of senescence-associated NAC transcription factors activated by ETHYLENE-INSENSITIVE2-mediated leaf senescence signalling in Arabidopsis. Journal of Experimental Botany. 2014;65(14):4023-36.

4. Sablowski RWM, Meyerowitz EM. A Homolog of NO APICAL MERISTEM Is an Immediate Target of the Floral Homeotic Genes APETALA3/PISTILLATA. Cell. 1998;92(1):93-103.

5. Guo Y, Gan S. AtNAP, a NAC family transcription factor, has an important role in leaf senescence. The Plant Journal. 2006;46(4):601-12.

6. Vogelmann K, Drechsel G, Bergler J, Subert C, Philippar K, Soll J, et al. Early Senescence and Cell Death in Arabidopsis saul1 Mutants Involves the PAD4-Dependent Salicylic Acid Pathway. Plant Physiology. 2012;159(4):1477-87.

7. Zipfel C, Robatzek S, Navarro L, Oakeley EJ, Jones JDG, Felix G, et al. Bacterial disease resistance in Arabidopsis through flagellin perception. Nature. 2004;428:764.

8. Hamel L-P, Benchabane M, Nicole M-C, Major IT, Morency M-J, Pelletier G, et al. Stress-Responsive Mitogen-Activated Protein Kinases Interact with the EAR Motif of a Poplar Zinc Finger Protein and Mediate Its Degradation through the 26S Proteasome. Plant Physiology. 2011;157(3):1379-93.

9. Jing L, Günter B, Tarja K, E. TP. WRKY70 modulates the selection of signaling pathways in plant defense. The Plant Journal. 2006;46(3):477-91.

10. Chen J, Nolan TM, Ye H, Zhang M, Tong H, Xin P, et al. Arabidopsis WRKY46, WRKY54, and WRKY70 Transcription Factors Are Involved in Brassinosteroid-Regulated Plant Growth and Drought Responses. The Plant Cell. 2017;29(6):1425-39.

11. Benschop JJ, Mohammed S, O'Flaherty M, Heck AJR, Slijper M, Menke FLH. Quantitative Phosphoproteomics of Early Elicitor Signaling in Arabidopsis. Molecular & Cellular Proteomics. 2007;6(7):1198-214.

12. Valon C, Smalle J, Goodman HM, Giraudat J. Characterization of an Arabidopsis thaliana gene (TMKL1) encoding a putative transmembrane protein with an unusual kinase-like domain. Plant Molecular Biology. 1993;23(2):415-21.
